# The microbiota-gut-brain axis perspective: mechanisms and intervention strategies for the comorbidity of chronic constipation and depression

**DOI:** 10.3389/fmicb.2026.1800520

**Published:** 2026-04-15

**Authors:** Bianfang Yu, Wen-wen Zhao, Longhao Tao, Kejian Li

**Affiliations:** 1 College of Traditional Chinese Medicine, Shandong University of Traditional Chinese Medicine, Jinan, China; 2 Department of Anorectal, Affiliated Hospital of Shandong University of Traditional Chinese Medicine, Jinan, China; 3 Health Management Center, Affiliated Hospital of Shandong University of Traditional Chinese Medicine, Jinan, China; 4 The First Clinical College, Shandong University of Traditional Chinese Medicine, Jinan, China

**Keywords:** chronic constipation, depression, gut dysbiosis, microbiota-gut-brain axis, psychobiotics, short-chain fatty acids

## Abstract

**Background:**

Chronic constipation and depression are highly prevalent worldwide. These two conditions frequently co-occur in clinical practice. Accumulating evidence indicates that gut microbiota dysbiosis mediates this comorbidity through the microbiota-gut-brain axis (MGBA).

**Methods:**

This narrative review systematically summarizes current research on MGBA bidirectional communication mechanisms, gut microbiota alterations in comorbid patients, and microbiota-targeted intervention strategies.

**Results:**

The MGBA facilitates bidirectional communication through four major pathways: neural pathways via the vagus nerve, immune pathways via cytokines, endocrine pathways via the HPA axis, and metabolic pathways via short-chain fatty acids and neurotransmitter precursors. Gut dysbiosis is associated with comorbidity and may contribute to its pathogenesis through multiple mechanisms. First, neurotransmitter metabolism becomes dysregulated, particularly in the serotonin and GABA systems. Second, chronic low-grade inflammation develops with elevated pro-inflammatory cytokines. Third, intestinal barrier dysfunction occurs, leading to increased permeability and bacterial translocation. Fourth, HPA axis hyperactivity emerges. Fifth, production of microbial metabolites is altered, including short-chain fatty acids and tryptophan metabolites. Comorbid patients exhibit characteristic microbiota signatures. These include reduced abundance of butyrate-producing bacteria such as Faecalibacterium, Roseburia, and Coprococcus. Microbial diversity decreases significantly. Pro-inflammatory taxa become enriched. Several evidence-based interventions show promise. These include psychobiotics, fecal microbiota transplantation, and dietary modifications such as Mediterranean diet and high-fiber intake. Exercise and integrative approaches including traditional Chinese medicine also demonstrate beneficial effects.

**Conclusion:**

The gut microbiota represents a critical hub connecting gastrointestinal and mental health. Microbiota-targeted therapies offer promising strategies for managing chronic constipation-depression comorbidity. Future research should establish causal relationships and develop reliable microbial biomarkers. Precision medicine approaches based on individual microbiome profiles are needed to optimize therapeutic outcomes.

## 1 Introduction

Chronic constipation and depression are highly prevalent health conditions worldwide, imposing substantial burdens on individual quality of life and healthcare systems. According to the Rome Criteria, the global prevalence of chronic constipation is approximately 14%, with annual healthcare expenditures in the United States alone amounting to billions of dollars (Rao et al., 2016). Concurrently, depression affects approximately 300 million people globally, with years lived with disability accounting for 37.3% of all mental disorders, rendering it a leading cause of disability (GBD 2019 Mental Disorders Collaborators, 2022). Notably, chronic constipation and depression frequently coexist in clinical practice. A large cohort study based on 449,459 participants from the UK Biobank demonstrated that patients with chronic constipation had a significantly elevated risk of developing depression (Yun et al., 2024). Research utilizing National Health and Nutrition Examination Survey (NHANES) data revealed that after adjusting for confounding factors including sex, age, body mass index, and smoking status, a significant positive association persisted between constipation and major depressive disorder (MDD) [odds ratio (OR) = 2.42, 95% confidence interval (CI): 1.86–3.14] (Wang et al., 2023). Furthermore, a 2024 bidirectional Mendelian randomization study confirmed the causal relationship between depression and chronic constipation genetically (OR = 11.43, 95% CI: 1.85–70.67, *P* = 0.008), while the reverse analysis did not identify a significant causal effect of constipation on depression, suggesting that depression may be an upstream factor in the development of chronic constipation (He et al., 2024).

The emergence of the microbiota-gut-brain axis (MGBA) theory in exploration of the pathophysiological mechanisms underlying the comorbidity of chronic constipation and depression has provided novel perspectives for understanding this complex phenomenon. The MGBA represents a bidirectional communication network between the central nervous system (CNS), enteric nervous system (ENS), and gut microbiota, transmitting signals through neural pathways (vagus nerve), endocrine pathways [hypothalamic-pituitary-adrenal (HPA) axis], immune pathways (cytokines, inflammatory mediators), and metabolic pathways [short-chain fatty acids (SCFAs), neurotransmitter precursors] (Cryan et al., 2019; Loh et al., 2024; Socala et al., 2021). The human gastrointestinal tract harbors trillions of microorganisms, whose collective genome far exceeds the coding capacity of the human genome. These microorganisms participate in nutrient metabolism and energy acquisition and are deeply involved in host immune system development, influencing the CNS structure and function by producing neurotransmitters, hormones, and metabolites (Sharon et al., 2016). Patients with neurodegenerative diseases such as Parkinson disease and Alzheimer disease exhibit altered gut microbiota during the preclinical stages (Schneider et al., 2024), suggesting that gut dysbiosis may be an early event in the pathogenesis of neuropsychiatric disorders.

Despite continuous improvements in treatment regimens for chronic constipation and depression, traditional therapeutic strategies continue to face numerous challenges. Conventional treatments for chronic constipation frequently demonstrate suboptimal long-term efficacy, with patients typically needing to try multiple medications before finding an effective regimen (Rao et al., 2016). Additionally, a considerable proportion of patients with depression respond inadequately to traditional antidepressant treatment and are classified as having treatment-resistant depression (Sforzini et al., 2022). More importantly, current treatment approaches predominantly target individual diseases, overlooking the complex interactions under comorbid conditions: many antidepressant medications affect gastrointestinal function and may exacerbate constipation symptoms (Yun et al., 2024). Baseline gut microbiota characteristics have been associated with treatment response in patients with depression, and systemic inflammation mediated by gut microbial dysbiosis may be important in treatment resistance (Xie et al., 2024). This evidence suggests that neglecting the gut microecological environment may be an important reason for suboptimal outcomes following traditional treatments.

Investigating comorbidity mechanisms from the MGBA perspective holds significant innovative value. Accumulating evidence indicates that gut dysbiosis participates in both the pathogenesis of constipation and the development of depression (Liu et al., 2023; Borrego-Ruiz and Borrego, 2024). Gut microbiota contributes to the development of functional constipation by interfering with intestinal barrier function, intestinal secretion, and intestinal motility (Li et al., 2024); simultaneously, dysbiosis-induced abnormalities in neurotransmitter metabolism, immune inflammation activation, and neuroendocrine dysregulation represent important mechanisms in depression pathogenesis (Kopera et al., 2024). This shared pathological basis may explain the high comorbidity rate between these two conditions. As a regulatable biological system, the gut microbiota provides novel intervention targets. Microecological therapies including probiotics, prebiotics, synbiotics, and fecal microbiota transplantation (FMT) may simultaneously improve constipation and depressive symptoms, breaking the vicious cycle of disease (Liu et al., 2023; Li et al., 2024). Specific probiotic supplementation not only improved bowel movement frequency and stool consistency in constipation patients, but also ameliorated mood and gastrointestinal symptoms in patients with depression (Luqman et al., 2024). Furthermore, specific microbial signatures may be biomarkers for disease risk assessment, early diagnosis, and therapeutic efficacy prediction. Constipation patients have altered abundances of specific bacterial genera (Li et al., 2024); depression patients demonstrate characteristic changes including decreased *Faecalibacterium* and increased *Actinobacteria* and Enterobacteriaceae (Lin et al., 2025).

MGBA-based research has provided innovative clinical intervention approaches for the comprehensive management of chronic constipation and depression comorbidity. This perspective promotes the development of integrative medicine models, emphasizing the intrinsic connection between somatic and psychiatric symptoms and suggesting the need for multidisciplinary collaboration between gastroenterology and psychiatry. Microecological interventions targeting gut dysbiosis typically demonstrate good safety and tolerability and can be complementary or alternative approaches to traditional treatments, particularly for patients resistant to conventional therapy (Liu et al., 2023; Simpson et al., 2021). Concurrently, early identification of gut dysbiosis may aid in preventing the development of chronic constipation and depression, providing early microecological intervention strategies for high-risk populations. In conclusion, investigating the comorbidity of chronic constipation and depression from the MGBA perspective would contribute to a deeper understanding of disease pathophysiology and provide scientific evidence for developing innovative diagnostic and therapeutic strategies, with the potential to contribute significantly to improving patient outcomes, enhancing quality of life, and reducing healthcare burdens.

## 2 Research progress on the MGBA

### 2.1 Overview of the MGBA

#### 2.1.1 Definition and structural components

**FIGURE 1:**
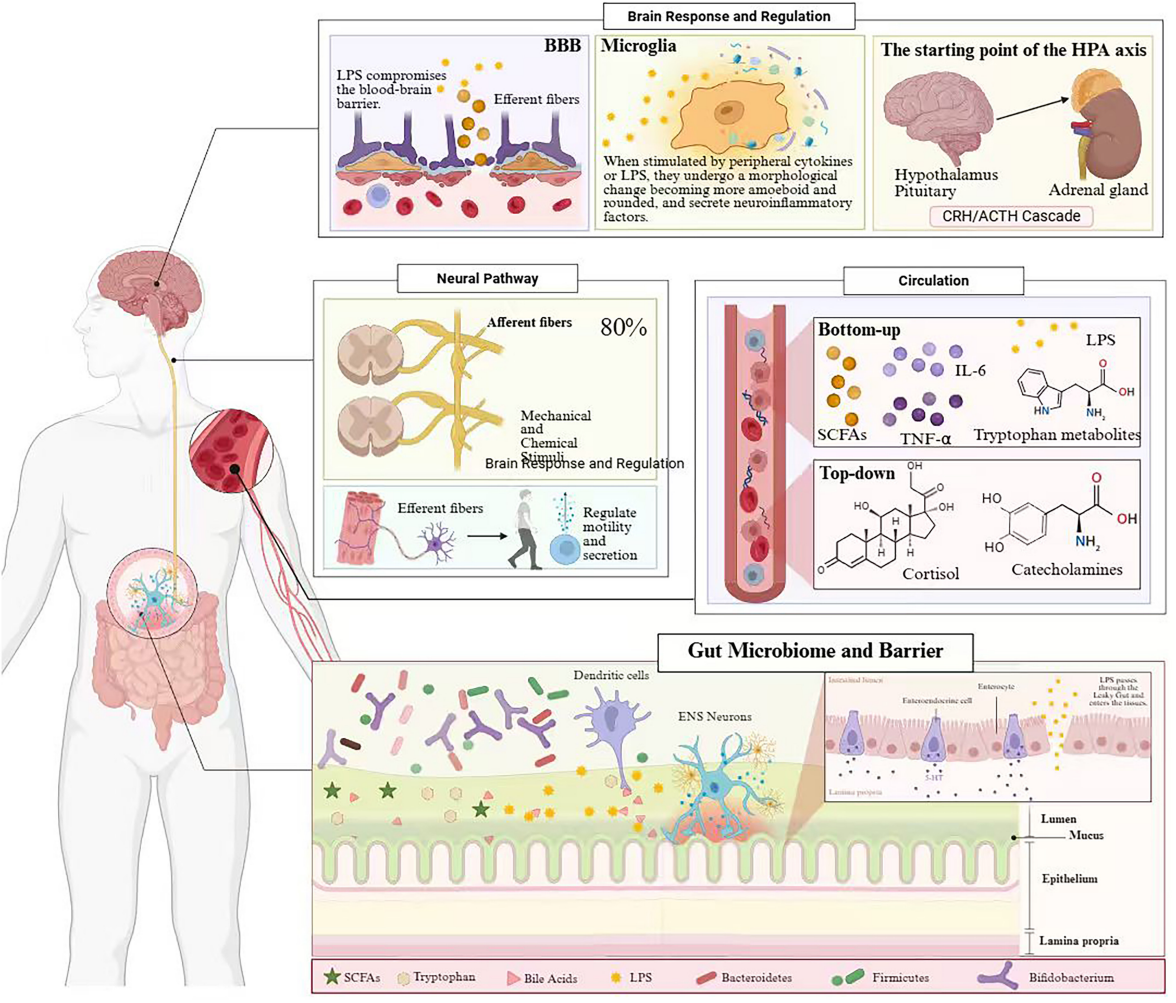
Schematic diagram of the bidirectional communication mechanisms within the MGBA. Solid arrows indicate pathways supported by multiple human studies or well-replicated preclinical evidence; dashed arrows indicate pathways proposed primarily on the basis of preclinical or limited human evidence. All depicted interactions represent plausible, evidence-informed hypotheses; this figure does not imply that the shown relationships have been causally established in human comorbid populations. This is an original schematic drawing.

The MGBA refers to a complex bidirectional communication network between the gut microbiota, the gastrointestinal tract with its ENS, and the CNS (Morais et al., 2021). Figure 1 demonstrates that the axis comprises several key structural components, including the gut microbiota (encompassing bacteria, fungi, viruses, and archaea), the intestinal epithelial barrier, the gut-associated immune system, the ENS, the vagus nerve, and the CNS (Cryan et al., 2019).

The gut microbiota represents a core element of the MGBA. Revised estimates have revealed that approximately 3.8 × 10^13^ bacteria colonize the human gastrointestinal tract, a number comparable to that of human somatic cells (approximately 3.0 × 10^13^), yielding a bacteria-to-human cell ratio of approximately 1.3:1 (Sender et al., 2016). These microorganisms are predominantly concentrated in the colon, forming a highly complex and dynamic ecosystem. The predominant phyla include Firmicutes, Bacteroidetes, Actinobacteria, and Proteobacteria, with Firmicutes and Bacteroidetes accounting for more than 90% of the total gut microbial population (Rinninella et al., 2019). Metagenomic studies have demonstrated substantial inter-individual variation in gut microbial composition and function, influenced by genetic background, dietary habits, lifestyle factors, medication use, and environmental exposures (Fan and Pedersen, 2021).

The intestinal epithelial barrier constitutes another critical MGBA component, comprising a single layer of columnar epithelial cells interconnected by tight junctions. This barrier exhibits selective permeability, permitting nutrient absorption while preventing the invasion of pathogens and harmful substances. Barrier integrity is maintained through multiple mechanisms, including mucus layer secretion, antimicrobial peptide production, and immunoglobulin A (IgA) secretion (Odenwald and Turner, 2017).

The ENS, frequently referred to as the “second brain,” contains approximately 200–600 million neurons distributed throughout the myenteric and submucosal plexuses of the intestinal wall (Furness et al., 2014). The ENS can independently regulate gut motility, secretion, and blood flow while communicating with the CNS through neurotransmitters, including serotonin (5-hydroxytryptamine, 5-HT), dopamine, and γ-aminobutyric acid (GABA) (Furness et al., 2014). Notably, more than 90% of the body’s 5-HT is produced in the gut, which provides a biological basis for understanding the relationship between mood disorders and gastrointestinal function (Yano et al., 2015).

The vagus nerve is the primary neural conduit connecting the gut and brain, and is important in information transmission within the MGBA. Afferent fibers, which constitute 80% of vagal nerve fibers, transmit mechanical, chemical, and immune signals from the gut to the CNS, whereas efferent fibers (20%) convey central commands to the gut, modulating digestive function and immune responses (Bonaz et al., 2018). Gut microbiota can influence brain function by modulating vagal nerve activity, and vagotomy experiments have confirmed the essential role of this pathway in microbiota-brain communication (Bravo et al., 2011).

#### 2.1.2 Bidirectional regulatory mechanisms

The bidirectional regulatory mechanisms of the MGBA are manifested through “bottom–up” pathways, whereby gut microbiota influence brain function, and “top–down” pathways, whereby the CNS modulates gut microecology (Mayer et al., 2022). This bidirectional communication ensures dynamic homeostasis within the internal environment and is important in stress responses, emotional regulation, and cognitive function.

In the bottom–up pathway, gut microbiota influence brain function through multiple mechanisms. First, microbial metabolites such as SCFAs can cross the blood–brain barrier (BBB) or activate peripheral receptors, directly or indirectly modulating neuronal activity and neuroinflammatory responses (Silva et al., 2020). Second, gut microbiota can synthesize and regulate the production of various neuroactive substances, including GABA, glutamate, norepinephrine, and acetylcholine (Strandwitz et al., 2019). Third, gut microbiota influence 5-HT and kynurenine generation by modulating the tryptophan metabolic pathways, affecting mood and cognitive function (Agus et al., 2018). The immune-mediated pathways are another important mechanism: gut microbiota regulate cytokine production by the intestinal immune system, such as interleukin-6 (IL-6), tumor necrosis factor-α (TNF-α), and IL-1β. These pro-inflammatory cytokines can influence brain function and neural plasticity through systemic circulation (Osadchiy et al., 2019).

The top–down regulatory mechanisms are mediated through neural, endocrine, and immune pathways by which the CNS influences gut microecology. The HPA axis is activated under stress conditions, and the released glucocorticoids can alter gut motility, secretion, and permeability, subsequently affecting gut microbial composition and function (Karl et al., 2018). Autonomic nervous system activity changes, particularly alterations in sympathetic and parasympathetic tone, can modulate intestinal blood flow, mucus secretion, and immune cell function, indirectly influencing the microbial ecosystem (Fung et al., 2017).

### 2.1.3 Key signaling pathways

Signal transmission within the MGBA involves multiple key pathways, including neural, immune, endocrine, and metabolic pathways, which interact to form a complex regulatory network.

Neural pathways: The vagus nerve is the primary neural connection, with afferent nerve fibers sensing mechanical stimuli, chemical signals, and microbial metabolites within the intestinal lumen, transmitting this information to the nucleus tractus solitarius (NTS), which subsequently projects to higher brain regions, including the amygdala, hippocampus, and prefrontal cortex (Bonaz et al., 2018). Experimental studies have demonstrated that probiotic intervention with Lactobacillus rhamnosus improved anxiety-like behavior and GABA receptor expression through vagus nerve-dependent mechanisms (Bravo et al., 2011). The detailed molecular mechanisms of vagal afferent signaling in the context of comorbidity are further discussed in section 4.5.

Immune pathways: The gut is the largest immune organ in the body, with gut-associated lymphoid tissue (GALT) representing approximately 70% of the entire immune system (Vighi et al., 2008). Gut microbiota activate the intestinal immune system through cell wall components such as lipopolysaccharide (LPS) and peptidoglycan, inducing the production of pro- or anti-inflammatory cytokines. These cytokines can reach the brain via systemic circulation, activating microglia and astrocytes and leading to neuroinflammatory responses (Erny et al., 2015). Impaired intestinal barrier function, resulting in “leaky gut,” allows bacterial metabolites and LPS to enter the systemic circulation, triggering systemic inflammatory responses closely associated with the pathogenesis of depression (Kelly et al., 2015).

Endocrine pathways: The HPA axis is the core endocrine system in stress responses. Under stress conditions, sequential release of corticotropin-releasing hormone (CRH) and adrenocorticotropic hormone (ACTH) ultimately increases cortisol secretion. Gut microbiota can modulate HPA axis activity through multiple mechanisms, including effects on HPA axis stress responsivity and programing during important developmental periods (Sudo et al., 2004).

Metabolic pathways: SCFAs, primarily including acetate (C2), propionate (C3), and butyrate (C4), are the main products of microbial fermentation of dietary fiber (Koh et al., 2016). SCFAs regulate immune function, intestinal barrier integrity, and nervous system activity by activating G protein-coupled receptors, including GPR41, GPR43, and GPR109A (Dalile et al., 2019). Furthermore, butyrate functions as a histone deacetylase (HDAC) inhibitor, regulating gene expression through epigenetic mechanisms (Donohoe et al., 2011).

### 2.2 Interactions between gut microbiota and the host

#### 2.2.1 Normal structure of the gut microecosystem

The gut microecosystem is a highly complex and dynamically balanced ecological system, and its normal structure is essential for maintaining host health. Healthy adults have a relatively stable gut microbiota composition, but it exhibits significant regional specificity across different intestinal segments (Kastl et al., 2020).

The Firmicutes/Bacteroidetes (F/B) ratio is commonly used as an indicator of gut microecological health status, and significant alterations in this ratio are associated with obesity, metabolic syndrome, and inflammatory diseases (Magne et al., 2020). Functional diversity is an important characteristic of the gut microbiota. Despite inter-individual variation in microbial species composition, the functional gene profile remains relatively conserved, and is termed “functional redundancy” (Human Microbiome Project Consortium, 2012).

#### 2.2.2 Definition and manifestations of dysbiosis

Gut dysbiosis refers to abnormal alterations in the gut microbiota composition, diversity, or function, disrupting the microecological balance (Levy et al., 2017). Dysbiosis encompasses multiple types of imbalance states: loss of microbial diversity, loss of core commensal bacteria, overgrowth of pathogens or opportunistic pathogens, and altered microbial function.

Dysbiosis associated with neuropsychiatric disorders has attracted widespread attention. Depression patients exhibit reduced gut microbial diversity, with a decreased abundance of SCFA-producing genera such as *Faecalibacterium* and *Coprococcus*, along with depletion of *Dialister* (Valles-Colomer et al., 2019). Parkinson disease patients have reduced SCFA-producing bacteria and increased LPS-producing Gram-negative bacteria in the gut, changes that may promote α-synuclein aggregation and neuroinflammation (Sampson et al., 2016).

#### 2.2.3 Physiological functions of microbial metabolites

Gut microbial metabolites are important mediators connecting microorganisms and their host. These small molecules function not only locally within the gut, but can also influence distant organs, including the brain, through systemic circulation (Koh et al., 2016). SCFAs are the primary products of microbial fermentation of dietary fiber, including acetate, propionate, and butyrate. Butyrate is the primary energy source for colonocytes, providing approximately 70% of their energy requirements, and is essential for maintaining intestinal barrier integrity (Roediger, 1980). In the CNS, SCFAs can cross the BBB, influencing microglial maturation and function and modulating neuroinflammatory responses (Erny et al., 2015). Tryptophan metabolites are important in neuropsychiatric function. Tryptophan is metabolized through three major pathways: the 5-HT pathway, the kynurenine pathway, and the indole pathway (Agus et al., 2018). The main MGBA components and signaling pathways are summarized in Table 1.

**TABLE 1:** Main MGBA components and signaling pathways.

MGBA component/signaling pathway	Structure, function, and mechanism	Key references
Gut microbiota (bacteria, fungi, viruses, archaea)	Core MGBA element; ∼3.8 × 10∧13 bacteria colonize gut (comparable to human somatic cells); predominantly Firmicutes and Bacteroidetes (> 90%); synthesizes neurotransmitters, metabolites (SCFAs, tryptophan catabolites); modulates host immune responses; collective genome far exceeds human genome coding capacity	(Cryan et al., 2019; Loh et al., 2024; Socala et al., 2021; Sender et al., 2016; Rinninella et al., 2019; Fan and Pedersen, 2021)
Intestinal epithelial barrier	Single layer of columnar epithelial cells interconnected by tight junction proteins (occludin, claudin, ZO-1); selective permeability for nutrient absorption while preventing pathogen invasion; integrity maintained by mucus layer secretion, antimicrobial peptide production, and IgA secretion	(Odenwald and Turner, 2017)
Enteric nervous system (ENS)—“second brain”	∼200–600 million neurons in myenteric and submucosal plexuses; independently regulates gut motility, secretion, and blood flow; communicates with CNS via neurotransmitters (5-HT, dopamine, GABA); > 90% of body’s 5-HT produced by intestinal EC cells—biological basis for mood-gut relationship	(Furness et al., 2014; Yano et al., 2015)
Vagus nerve	Primary neural conduit connecting gut and brain; 80% afferent fibers (gut-to-brain: mechanical, chemical, immune signals) + 20% efferent fibers (brain-to-gut: digestive function, immune responses); signals project to nucleus tractus solitarius (NTS), then to amygdala, hippocampus, prefrontal cortex; vagotomy abolishes microbiota-brain communication	(Bonaz et al., 2018; Bravo et al., 2011)
Neural pathway (vagal signaling)	Microbial metabolites, 5-HT released by EC cells, and bacterial products activate vagal afferent nerve endings; signals transmitted to NTS then to locus coeruleus, dorsal raphe nucleus, amygdala, hippocampus; L. rhamnosus JB-1 improved anxiety-like behavior and GABA receptor expression via vagus nerve-dependent mechanism; vagotomy completely abolished probiotic psychotropic effects	(Bonaz et al., 2018; Bravo et al., 2011; Bercik et al., 2011)
Immune pathway (cytokine signaling)	GALT represents ∼70% of entire immune system; gut microbiota activate intestinal immune system via LPS and peptidoglycan (TLR4 signaling), inducing pro-inflammatory cytokines (IL-6, TNF-alpha, IL-1 beta); cytokines reach brain via systemic circulation, activating microglia/astrocytes leading to neuroinflammation; “leaky gut” allows LPS/bacterial products into systemic circulation—closely linked to depression pathogenesis	(Vighi et al., 2008; Erny et al., 2015; Kelly et al., 2015; Miller and Raison, 2016)
Endocrine pathway (HPA axis)	Under stress: sequential CRH - > ACTH release - > cortisol secretion; gut microbiota modulate HPA axis reactivity and programing during critical developmental windows; germ-free mice exhibit 2–3x elevated corticosterone vs. conventional mice; colonization within 14 days post-birth reverses HPA hyperreactivity; cortisol alters gut motility, barrier integrity, and microbiota composition	(Sudo et al., 2004; Foster et al., 2017; Juruena et al., 2018)
Metabolic pathway (short-chain fatty acids—SCFAs)	Acetate (C2), propionate (C3), butyrate (C4)—primary products of microbial fermentation of dietary fiber; activate G protein-coupled receptors GPR41, GPR43, GPR109A; butyrate acts as HDAC inhibitor (epigenetic regulation), serves as primary colonocyte energy source (∼70% energy requirements), crosses BBB, upregulates BDNF, promotes microglial maturation, activates GPR41/43 on EC cells to stimulate 5-HT release and initiate peristaltic reflexes	(Koh et al., 2016; Dalile et al., 2019; Koh et al., 2016; Dalile et al., 2019; Donohoe et al., 2011; Silva et al., 2020)
Metabolic pathway (tryptophan metabolites)	Tryptophan metabolized via three pathways: (1) 5-HT pathway (only 1–2% of Trp); (2) kynurenine pathway (∼95% of Trp; IDO/TDO activated by pro-inflammatory cytokines - > kynurenine - > QUIN [neurotoxic, NMDA agonist, in microglia] or KYNA [neuroprotective, in astrocytes]); (3) microbial indole pathway; gut microbiota regulate flux among all three pathways; overactivation of kynurenine pathway depletes Trp available for 5-HT synthesis	(Agus et al., 2018; O’Mahony et al., 2015; Cervenka et al., 2017; Schwarcz et al., 2012)

CNS, central nervous system; ENS, enteric nervous system; EC, enterochromaffin; GALT, gut-associated lymphoid tissue; HPA, hypothalamic-pituitary-adrenal; SCFAs, short-chain fatty acids; 5-HT, serotonin; GABA, gamma-aminobutyric acid; BBB, blood-brain barrier; NTS, nucleus tractus solitarius; LPS, lipopolysaccharide; IDO, indoleamine 2,3-dioxygenase; TDO, tryptophan 2,3-dioxygenase; QUIN, quinolinic acid; KYNA, kynurenic acid; HDAC, histone deacetylase; BDNF, brain-derived neurotrophic factor; CRH, corticotropin-releasing hormone; ACTH, adrenocorticotropic hormone.

## 3 Comorbid characteristics of chronic constipation and depression

### 3.1 Epidemiological associations

#### 3.1.1 Comorbidity prevalence data

Multiple large-scale epidemiological studies have confirmed that a significant bidirectional association exists between chronic constipation and depression. Chronic constipation, defined as persistent symptoms of difficult defecation, reduced bowel movement frequency, or incomplete evacuation lasting at least 3 months, has a global prevalence of approximately 14–15%, with considerable variation across regions and populations (Bharucha and Lacy, 2020). It should be noted that constipation definitions vary across cited studies—some apply Rome IV criteria, others use Rome III, self-report, or ICD-based diagnoses. This definitional heterogeneity may contribute to variability in reported comorbidity rates and microbiota findings.

Depression is one of the most common psychiatric disorders and has a global prevalence of approximately 4.4% and demonstrates an increasing trend (GBD 2019 Mental Disorders Collaborators, 2022). Depression diagnoses in cited studies were variously established by DSM-IV/5 structured interview (e.g., Zheng et al., Caso et al.), validated rating scales (PHQ-9, HAMD, HADS), or self-report, which introduces diagnostic heterogeneity that limits cross-study comparisons.

**FIGURE 2:**
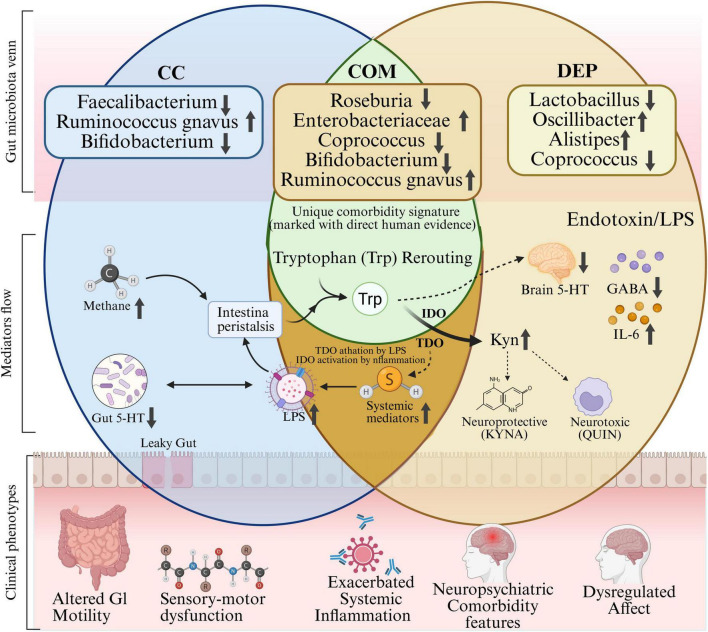
Specific gut microbiota alterations in the comorbid state of chronic constipation and depression. Venn diagram of gut microbiota alterations and mechanistic mediators in chronic constipation (CC), depression (DEP), and their comorbid state (COM). Microbiota alterations shown in the CC and DEP zones are derived from single-disease cohort studies; entries in the COM zone are supported by evidence from studies in which both conditions co-occur or have been directly cross-referenced across independent cohorts (indicated by the annotation “Unique comorbidity signature—marked with direct human evidence”). ↑ and ↓ indicate increased and decreased relative abundance, respectively, compared to healthy controls. In the Mediators flow layer, solid arrows indicate relationships with support from multiple human or well-replicated experimental studies; dashed arrows indicate pathways inferred primarily from preclinical or mechanistic data (e.g., IDO/TDO activation by systemic LPS and inflammation leading to tryptophan rerouting away from serotonin synthesis). This figure is an original schematic and does not imply that all depicted interactions have been causally established in human comorbid populations.

Notably, the comorbidity rate between these two conditions far exceeds what would be expected by chance, suggesting intimate pathophysiological connections. Figure 2 details the mechanism of their mutual reinforcement.

Multiple large-scale, cross-sectional studies have confirmed the significant association between chronic constipation and depression. A study based on the NHANES 2005–2010 that involved 12,352 adult participants demonstrated that the prevalence of MDD in constipated patients was 20.9%, significantly higher than the 7.9% in non-constipated individuals (*P* < 0.001) (Wang et al., 2023). Another NHANES-based study (2009–2010) determined that the prevalence of chronic constipation in depressed individuals was 9.10% [95% confidence interval (CI): 7.02–11.69%], significantly higher than the 6.55% (95% CI: 5.55–7.70%, *P* = 0.003) in non-depressed individuals (Ballou et al., 2019). Similarly, a cross-sectional study from Iran that involved 4,763 adults demonstrated that depressed individuals had a significantly increased risk of constipation, with an adjusted odds ratio (OR) of 1.69 (95% CI: 1.37–2.09) (Adibi et al., 2022).

Longitudinally, bidirectional temporal relationships exist between chronic constipation and depression. A large population-based cohort study published in 2024 demonstrated that both diagnosed and self-reported constipation were prospectively associated with an elevated risk of depression, suggesting that constipation may be an independent risk factor or prodromal symptom of depression (Yun et al., 2024). Another 12-year prospective population-based study demonstrated that the brain–gut pathway is bidirectional: elevated baseline anxiety levels predicted new-onset functional gastrointestinal disorders 12 years later, while individuals with functional gastrointestinal disorders at baseline exhibited significantly elevated anxiety and depression levels at follow-up (Koloski et al., 2012). These findings suggest the existence of a mutually reinforcing vicious cycle between chronic constipation and depression.

Comorbidity severity demonstrates a dose–response relationship. Constipation symptom severity correlates positively with depression symptom severity (Drossman and Hasler, 2016). Patients diagnosed with functional constipation using the Rome IV Criteria demonstrate high rates of comorbid mood disorders. Subtype-specific comorbidity patterns deserve attention, with slow transit constipation (STC) patients demonstrating significantly higher depression prevalence compared to outlet obstruction constipation (OOC) patients (Knowles et al., 1999). This finding suggests that different pathophysiological mechanisms of constipation are associated with varying strengths of association with psychological disorders. Furthermore, comorbid patients present with more complex clinical manifestations, significantly impaired quality of life, increased treatment difficulty, and higher healthcare resource consumption.

### 3.1.2 Clinical manifestations of mutual influence

The comorbidity of chronic constipation and depression leads to complex clinical presentations, with the two conditions mutually influencing each other to form a vicious cycle that significantly impairs quality of life.

In patients with chronic constipation, the presence of depressive symptoms renders constipation symptoms more refractory and difficult to treat. Constipated patients with comorbid depression exhibit more severe bloating, abdominal pain, and sense of incomplete evacuation, with lower defecation frequency and harder stool consistency (Koloski et al., 2002). Studies that used the Patient Assessment of Constipation Symptoms (PAC-SYM) scale have demonstrated that constipated patients with comorbid psychological disorders scored significantly higher than those with constipation alone (Frank et al., 1999). Furthermore, these patients exhibit a poorer response to conventional treatments, requiring longer treatment durations and more complex therapeutic regimens (Wald et al., 2007).

Depressive symptoms in constipated patients have specific characteristics. Such patients more commonly present with somatic symptoms including fatigue, sleep disturbances, appetite changes, and pain, while traditional emotional symptoms such as sadness and hopelessness may be relatively mild (Ballou and Keefer, 2017). This “somatized depression” renders diagnosis more challenging and is easily overlooked by clinicians. Furthermore, constipation-related discomfort and anxiety exacerbate depressive symptoms, forming a vicious psychological-somatic symptom cycle.

In depression patients, the occurrence of constipation is frequently misattributed to antidepressant medication side effects. In reality, constipation incidence is significantly higher even in depression patients not receiving pharmacological treatment than in healthy controls (Mawe and Hoffman, 2013). Antidepressant medications, particularly tricyclic antidepressants (TCAs) and selective 5-HT reuptake inhibitors (SSRIs), may exacerbate constipation, but constipation may also represent a somatic symptom of depression.

Comorbid patients’ quality of life suffers a dual effect. An assessment using the SF-36 Health Survey revealed that comorbid patients scored significantly lower across all dimensions compared to patients with single conditions and healthy controls, particularly in physical functioning, bodily pain, vitality, and social functioning (Gracie et al., 2019). Physical discomfort and social limitations from constipation symptoms, combined with low mood and anhedonia from depressive symptoms, result in severe functional impairment. Sleep disturbance is an important bridge connecting constipation and depression, with research indicating that most comorbid patients experience sleep quality issues including difficulty initiating sleep, early awakening, and sleep fragmentation (Vege et al., 2004). Increased pain sensitivity is another prominent feature in comorbid patients, with visceral hypersensitivity being key in this process, as central and peripheral sensitization together lead to pain symptom amplification (Whitehead et al., 2002).

#### 3.1.3 High-risk population characteristics

Certain populations carry an elevated risk for chronic constipation and depression comorbidity, and identifying these high-risk groups is important for early prevention and intervention.

Gender differences represent one of the most significant demographic characteristics, with females demonstrating higher prevalence rates for both chronic constipation and depression, along with significantly increased comorbidity risk (Kim and Kim, 2018). This gender disparity may relate to multiple factors including sex hormone levels, differences in gut motility, psychosocial factors, and healthcare-seeking behavior. Reproductive-age women, particularly those who have experienced multiple pregnancies, face a higher comorbidity risk due to hormonal fluctuations and pelvic floor function changes. Emerging evidence suggests that gut microbiota composition differs between sexes, with females showing distinct Firmicutes/Bacteroidetes ratios and SCFA-producing taxa profiles compared to males. Given the female predominance in both chronic constipation and depression, future studies should consider sex-stratified microbiota analyses.

Age significantly influences comorbidity patterns. The elderly population (≥ 65 years) has a significantly elevated chronic constipation prevalence, commonly accompanied by multiple chronic diseases, polypharmacy, and cognitive function decline, rendering clinical management more complex (Gallegos-Orozco et al., 2012). Furthermore, socioeconomic status is closely associated with comorbidity risk, with lower-income, lower-educational attainment, and unemployed individuals demonstrating an increased susceptibility to chronic constipation and depression comorbidity (Howell et al., 2004). This may be related to poor nutritional status, limited healthcare access, increased life stress, and lower health literacy.

Medical history is an important predictor of comorbidity. Patients with histories of irritable bowel syndrome (IBS), inflammatory bowel disease (IBD), or other gastrointestinal disorders face a significantly increased comorbidity risk (Gracie et al., 2018). Similarly, patients with histories of anxiety disorders, post-traumatic stress disorder (PTSD), or other psychiatric disorders also belong to high-risk groups. Adverse childhood experiences (ACEs), including abuse, neglect, and family dysfunction, have a dose–response relationship with adult comorbidity risk (Park et al., 2016).

Lifestyle factors significantly influence comorbidity risk, with individuals who have insufficient dietary fiber intake, inadequate fluid intake, lack of regular exercise, and poor sleep quality demonstrating a notably increased comorbidity risk (Staudacher and Whelan, 2017). Medication use is another important risk factor, with patients on long-term opioids, anticholinergic agents, calcium channel blockers, and antidepressants demonstrating a significantly increased constipation incidence (Coyne et al., 2014). Additionally, genetic susceptibility should not be overlooked, with twin studies indicating that genetic factors contribute approximately 30–40% to comorbidity, and certain genetic polymorphisms such as 5-HT transporter gene (*SLC6A4*) and brain-derived neurotrophic factor (*BDNF*) gene variants are associated with comorbidity susceptibility (Levy et al., 2001).

### 3.2 Pathophysiological connections

### 3.2.1 Gut dysmotility

Gut dysmotility represents an important pathophysiological mechanism connecting chronic constipation and depression. Normal gut motility depends on coordinated control involving smooth muscle contraction, ENS regulation, hormone secretion, and CNS input (Furness, 2012). In patients with chronic constipation and depression comorbidity, these regulatory mechanisms all demonstrate varying degrees of dysfunction.

Prolonged intestinal transit time is a core feature of chronic constipation, particularly pronounced in comorbid patients. Studies that used wireless motility capsules have reported significantly prolonged colonic transit times in STC patients (Rao et al., 2011). This transit delay primarily occurs in the left colon and rectosigmoid region, closely related to autonomic nervous system dysfunction and ENS alterations associated with depression. Depression patients exhibit reduced parasympathetic activity and relative sympathetic hyperactivity, and this autonomic imbalance inhibits the generation and propagation of propulsive intestinal contractions (Bonaz et al., 2017). High-resolution colonic manometry studies have demonstrated that chronic constipation patients exhibit significantly reduced frequency of high-amplitude propagating contractions (HAPCs), decreasing from normal levels of 6–10 per 24 h to 2–4 per 24 h (Dinning et al., 2015). HAPCs are the primary driving force for distal movement of intestinal contents, and reductions in their frequency and amplitude directly contribute to constipation symptoms.

Interstitial cells of Cajal (ICC) dysfunction represents the cellular basis of gut dysmotility. As intestinal pacemaker cells, ICC generate slow wave potentials and coordinate rhythmic smooth muscle contractions. STC patients demonstrate a 40–100% reduction in colonic ICC volume with morphological abnormalities (He et al., 2000). Mechanisms that include oxidative stress, inflammatory factors, and neurotrophic factor deficiency may mediate this ICC injury. BDNF is significantly decreased in depression patients, and BDNF is essential for ICC survival and function (Coulie et al., 2000).

Structural and functional alterations in the ENS represent key mechanisms underlying gut dysmotility in comorbid patients. The ENS contains approximately 500 million neurons, forming the myenteric and submucosal plexuses and regulating intestinal motility through multiple neurotransmitters. Chronic constipation patients have reduced ENS neuron numbers, decreased nerve fiber density, and abnormal neurotransmitter expression (De Giorgio et al., 2004).

Neurotransmitter system imbalance is central in gut dysmotility. 5-HT is a key neurotransmitter regulating gut motility, with 95% of body 5-HT produced by enterochromaffin (EC) cells. Chronic constipation patients have reduced EC cell density, decreased 5-HT synthesis, and significantly lowered colonic tissue 5-HT concentrations (Coates et al., 2004). Intestinal 5-HT signal transduction in depression patients is abnormal due to long-term SSRI use or the disease, with altered 5-HT3 and 5-HT4 receptor expression and function, further exacerbating gut dysmotility (Gershon, 2004).

#### 3.2.2 Neuroendocrine alterations

Neuroendocrine system dysregulation represents an important pathophysiological mechanism in chronic constipation and depression comorbidity, involving abnormalities in the HPA axis, hypothalamic-pituitary-thyroid (HPT) axis, and various gastrointestinal hormones (Konturek et al., 2011).

HPA axis hyperactivity is a core neuroendocrine feature of depression and significantly affects gut function. In depression patients, impaired HPA axis negative feedback regulation leads to sustained elevation of corticotropin-releasing hormone (CRH) and cortisol (Pariante and Lightman, 2008). Elevated cortisol levels affect gastrointestinal function through several mechanisms: inhibiting gut motility, reducing intestinal blood flow, impairing gut barrier integrity, altering gut microbiota composition, and increasing visceral hypersensitivity (Vanuytsel et al., 2014).

The peripheral effects of CRH are important in gut–brain interactions. Beyond central effects, CRH and its receptors (CRH-R1 and CRH-R2) are also widely expressed in the gut. Under stress conditions, CRH influences gut motility and secretory function by activating intestinal mast cells and ENS neurons (Larauche et al., 2009).

Thyroid dysfunction is also important in chronic constipation and depression comorbidity. Subclinical hypothyroidism (elevated thyroid-stimulating hormone with normal thyroid hormones) prevalence is increased in comorbid patients (Duntas and Maillis, 2013). Thyroid hormones promote intestinal motility, and their deficiency leads to prolonged intestinal transit time. Meanwhile, hypothyroidism is associated with depressive symptoms, and the two may mutually reinforce each other.

Gastrointestinal hormone dysregulation represents an important endocrine pathway connecting the gut and brain. Motilin is an important hormone promoting gastrointestinal motility whose plasma levels are decreased in comorbid patients, which are closely related to weakened gut motility and defecation difficulties (Sjölund et al., 1996). Ghrelin not only regulates appetite and energy metabolism, but also promotes gastrointestinal motility. Fasting ghrelin levels are decreased in depression patients, while chronic constipation patients also demonstrate abnormal ghrelin secretion, where these changes are more pronounced in comorbidity (Kluge et al., 2007). Furthermore, melatonin system dysregulation participates in the pathophysiology of comorbidity. Melatonin not only regulates circadian rhythm and sleep, but is also abundantly produced in the gut, regulating intestinal motility and barrier function. Melatonin supplementation may simultaneously improve sleep, mood, and constipation symptoms (Song et al., 2005).

#### 3.2.3 Immune-inflammatory responses

Immune system dysregulation and chronic low-grade inflammation represent important pathophysiological mechanisms in chronic constipation and depression comorbidity. Increasing evidence indicates that both conditions are associated with immune activation and pro-inflammatory states (Maes et al., 2008).

Elevated pro-inflammatory cytokines are a prominent feature in comorbid patients. Systematic meta-analyses have demonstrated that depression patients have significantly elevated serum IL-6, TNF-α, and C-reactive protein (CRP) levels (Osimo et al., 2020). Chronic constipation patients, particularly those with STC, also demonstrate increased pro-inflammatory cytokine levels (Barbara et al., 2004).

The dual role of IL-6 deserves particular attention. Peripheral IL-6 can enter the CNS by activating vagal afferent fibers, crossing BBB vulnerable regions (such as circumventricular organs), or through endothelial cell transport, activating microglia and astrocytes to induce neuroinflammation (Hodes et al., 2015). TNF-α acts through several mechanisms, directly affecting neuronal function, reducing 5-HT synthesis, impairing gut barrier integrity, and promoting intestinal muscularis inflammation.

Local gut immune activation is important in chronic constipation. Histopathological studies have demonstrated increased numbers of CD3 + T lymphocytes, CD68 + macrophages, and mast cells in the colonic mucosa and muscularis of chronic constipation patients (Törnblom et al., 2002). Inflammatory mediators released by these immune cells, including cytokines, chemokines, histamine, and tryptase, directly damage ENS neurons and ICC, leading to gut dysmotility.

Gut barrier dysfunction and increased intestinal permeability are prevalent in comorbid patients. The expression of intestinal epithelial tight junction proteins (such as occludin, claudin, and ZO-1) is decreased, and intestinal permeability increases, leading to the entry of bacterial products such as LPS, peptidoglycan, and undigested food antigens into the bloodstream (Camilleri and Gorman, 2007). Elevated plasma LPS levels activate Toll-like receptor 4 (TLR4) signaling pathways, triggering systemic inflammatory responses, termed “metabolic endotoxemia” (Cani et al., 2007).

Oxidative stress and antioxidant system imbalance are important components of immune-inflammatory responses. Depression patients have elevated levels of oxidative stress markers such as malondialdehyde (MDA) and 8-hydroxy-2′-deoxyguanosine (8-OHdG), while antioxidant enzymes such as superoxide dismutase (SOD) and glutathione peroxidase (GPx) exhibit decreased activity (Maes and Twisk, 2011). Furthermore, colonic tissue from chronic constipation patients demonstrates oxidative stress damage, with increased reactive oxygen species (ROS) production, mitochondrial dysfunction, and increased apoptosis (Collins and Bercik, 2009). Oxidative stress damages ENS neurons and ICC, exacerbating gut dysmotility while simultaneously promoting depressive symptoms by activating inflammatory pathways and neuronal damage.

Kynurenine pathway activation is an important metabolic pathway connecting inflammation and depression. Indoleamine 2,3-dioxygenase (IDO) and tryptophan 2,3-dioxygenase (TDO) are activated under pro-inflammatory conditions, directing more tryptophan metabolism toward kynurenine rather than 5-HT (O’Mahony et al., 2015). Kynurenine is metabolized in astrocytes and microglia to quinolinic acid (QUIN, an NMDA receptor agonist with neurotoxic properties) or kynurenic acid (KYNA, a neuroprotective metabolite). The QUIN/KYNA ratio increases under chronic inflammatory conditions, leading to neuronal damage, glutamatergic system dysregulation, and depressive symptoms (Miller and Raison, 2016). Comorbid patients have significantly elevated plasma kynurenine/tryptophan ratios, positively correlated with both constipation severity and depression scores (Myint and Kim, 2014). The key epidemiological and clinical studies on chronic constipation-depression comorbidity are summarized in Table 2.

**TABLE 2:** Key epidemiological and clinical studies on chronic constipation-depression comorbidity (section 3).

References	Study design	Population	Diagnostic criteria	Main findings	Clinical implications
Wang et al. (2023)	Cross-sectional	12,352 US adults (NHANES 2005–2010)	Self-report constipation; DSM-IV MDD	MDD prevalence 20.9% in constipated vs. 7.9% in non-constipated (*P* < 0.001); OR = 2.42 (95% CI: 1.86–3.14) after adjustment for sex, age, BMI, smoking	Robust association between constipation and MDD in large representative US sample; highlights need for psychiatric screening in constipated patients
Ballou et al. (2019)	Cross-sectional	NHANES 2009–2010 adults	DSM-IV depression; self-report constipation	Constipation prevalence 9.10% (95% CI: 7.02–11.69%) in depressed vs. 6.55% (95% CI: 5.55–7.70%) in non-depressed (*P* = 0.003)	Bidirectional association confirmed; depression associated with significantly increased constipation prevalence in nationally representative sample
Adibi et al. (2022)	Cross-sectional	4,763 Iranian adults	Validated depression scale; Rome criteria constipation	Adjusted OR = 1.69 (95% CI: 1.37–2.09) for constipation risk in depressed individuals	Association persists across different populations and diagnostic criteria; cross-cultural evidence strengthens comorbidity findings
Yun et al. (2024)	Prospective cohort (longitudinal)	449,459 UK Biobank participants	ICD-based depression; diagnosed and self-reported constipation	Both diagnosed and self-reported constipation prospectively associated with elevated depression risk; constipation may be independent risk factor or prodromal symptom	Largest cohort providing longitudinal evidence for constipation - > depression temporal relationship; strengthens causal inference in one direction
Koloski et al. (2012)	12-year prospective cohort (bidirectional)	Population-based sample, 12-year follow-up	Validated anxiety/depression scales; Rome criteria FGIDs	Elevated baseline anxiety predicted new-onset FGIDs at 12 years; FGIDs at baseline predicted significantly elevated anxiety and depression at follow-up	Bidirectional brain-gut pathway confirmed over 12 years; establishes mutually reinforcing vicious cycle between gastrointestinal and psychiatric symptoms
He et al. (2024)	Bidirectional Mendelian Randomization	NHANES 2005–2010 + genome-wide data	Genetic instruments for depression and constipation (SNP-based)	Depression causally associated with constipation (OR = 11.43, 95% CI: 1.85–70.67, *P* = 0.008); reverse direction (constipation - > depression) not statistically significant	Genetic evidence supporting depression as upstream causal factor for constipation; strongest causal inference methodology available for observational data; direction of causality partially established
Knowles et al. (1999)	Clinical case-control	STC vs. OOC patients	Rome criteria subtypes; validated depression scales	STC patients showed significantly higher depression prevalence compared to OOC patients	Constipation subtype differences in psychiatric comorbidity; STC (autonomic/ENS dysfunction) more closely linked to neuropsychiatric symptoms than OOC (mechanical/structural)
Osimo et al. (2020)	Systematic meta-analysis	5,166 depression patients + 5,083 controls (pooled)	DSM-based depression; CRP, IL-6, TNF-alpha measurement	Depression patients had significantly elevated IL-6, TNF-alpha, and CRP levels vs. controls; consistent across studies	Chronic low-grade systemic inflammation is a reproducible biological feature of depression; provides immunological bridge to gut dysbiosis and constipation pathophysiology
Valles-Colomer et al. (2019)	Cross-sectional + validation cohort	1,054 (Flemish Gut Flora) + 1,070 (validation)	DSM-based depression equivalents; quality-of-life scales; 16S microbiota profiling	Coprococcus and Dialister depleted in depression patients even after adjusting for antidepressant use; butyrate-producing Faecalibacterium and Coprococcus positively correlated with quality of life	Large-scale population microbiota study directly linking specific taxa to depression and quality of life; antidepressant-controlled design strengthens findings; establishes SCFA producers as candidate biomarkers

CC, chronic constipation; MDD, major depressive disorder; NHANES, National Health and Nutrition Examination Survey; OR, odds ratio; CI, confidence interval; FGID, functional gastrointestinal disorder; STC, slow transit constipation; OOC, outlet obstruction constipation; HC, healthy controls; DSM, Diagnostic and Statistical Manual; ICD, International Classification of Diseases; SNP, single nucleotide polymorphism. Diagnostic criteria varied across studies—some applied Rome IV/III criteria, others used ICD-based diagnoses, validated rating scales (PHQ-9, HAMD), or self-report. This definitional heterogeneity may contribute to variability in reported comorbidity rates and should be considered when comparing effect sizes across studies.

## 4 Mediation of comorbidity

It is important to note that the mechanistic pathways described below are largely inferred from preclinical models, *in vitro* experiments, and observational human studies. Direct causal evidence in human comorbid populations remains limited, and the pathways should be interpreted as plausible, evidence-informed hypotheses rather than established mechanisms. Gut microbiota dysbiosis mediates the comorbidity of chronic constipation and depression through several intertwined molecular pathways. These mechanisms encompass neurotransmitter metabolism, immune-inflammatory responses, neuroendocrine regulation, and metabolite signaling, collectively forming the molecular basis of the MGBA. A thorough understanding of these mechanisms would not only aid the elucidation of the pathophysiological nature of comorbidity but would also provide theoretical foundations for developing microbiome-targeted therapeutic strategies.

### 4.1 Neurotransmitter metabolic pathways

Abnormal neurotransmitter metabolism represents the core molecular link connecting the gut microbiota with CNS function. Gut microorganisms not only directly synthesize various neuroactive substances, but also indirectly influence neurotransmitter biosynthesis and metabolism by regulating the expression and activity of host neurotransmitter-synthesizing enzymes. Figure 2 delineates how the aforementioned factors interact through the gut–brain axis, including the core molecular regulatory mechanisms within the MGBA.

#### 4.1.1 The 5-HT system

The 5-HT system plays a central role in the pathophysiology of chronic constipation–depression comorbidity. Approximately 95% of the body’s 5-HT is synthesized by intestinal EC cells (Yano et al., 2015), a finding that repositioned the intestine as a key neuroendocrine organ.

At the biosynthetic level, butyrate upregulates TPH1 expression by enhancing histone acetylation at the TPH1 gene promoter region, thereby promoting 5-HT production (Yano et al., 2015; Dalile et al., 2019). The broader roles of SCFAs in gut–brain communication are detailed in section 4.4.

In comorbid patients, dysbiosis upregulates SERT expression and impairs EC cell function, reducing colonic 5-HT content (Cao et al., 2017). Reduced 5-HT slows intestinal peristalsis by diminishing activation of 5-HT4 receptors in the myenteric plexus. Overactivation of 5-HT3 receptors contributes to visceral hypersensitivity, while functional downregulation of 5-HT4 receptors is directly linked to constipation. More than 60% of patients on long-term SSRIs report constipation (Cryan et al., 2019), suggesting that antidepressant treatment may worsen gastrointestinal symptoms by altering peripheral 5-HT signaling.

Carriers of the short allele of the SERT gene polymorphism (5-HTTLPR) show lower transporter activity and greater susceptibility to comorbid phenotypes (Cao et al., 2017). Although peripheral 5-HT cannot cross the BBB directly, it influences CNS function indirectly by activating vagal afferent fibers, modulating immune cells, and affecting tryptophan availability (Agirman et al., 2021).

#### 4.1.2 The GABA pathway

Beyond the 5-HT system, GABA, as the most abundant inhibitory neurotransmitter in the CNS, has a core role in mood regulation and anxiety control. Recent research has determined that gut microorganisms not only produce GABA, but that the intestinal GABA system plays a non-negligible role in regulating gut motility and immune function. Strandwitz et al. (2019) systematically identified the diversity of GABA-producing bacteria in the human gut, finding that *Bacteroides* species can produce substantial quantities of GABA. Additionally, *Lactobacillus* and *Bifidobacterium* have been confirmed to convert glutamate to GABA through glutamate decarboxylase (GAD). An important finding of this study was that fecal *Bacteroides* abundance in depression patients was negatively correlated with functional connectivity in depression-associated brain regions, providing clear therapeutic targets for microbiota-directed interventions.

Intestinal GABA exerts biological effects through two types of receptors: ionotropic GABA_A receptors and metabotropic GABA_B receptors. Seifi et al. (2018) detailed the differential roles of different GABA receptor subtypes in stress-induced colonic inflammation, and revealed a complex interactive network between the GABA system and the 5-HT system: approximately 30% of enteric neurons simultaneously express both neurotransmitters. From a central perspective, magnetic resonance spectroscopy studies have demonstrated that prefrontal cortex GABA concentration decreases by 18–22% in depression patients, which was significantly and positively correlated to gut GABA-producing microbial abundance (Cryan et al., 2019), suggesting that peripheral microbial composition may reflect or influence central neurotransmitter levels.

Probiotic intervention studies have provided direct evidence for the causal role of the GABA pathway in comorbidity. Bravo et al. (2011) demonstrated that administering *Lactobacillus rhamnosus* JB-1 to mice significantly increased GABA_B receptor expression in the hippocampus and cortex and improved depression- and anxiety-like behaviors, while simultaneously improving gut motor function. More compellingly, vagotomy completely abolished these beneficial effects, demonstrating for the first time at the molecular level the necessity and sufficiency of the vagus nerve in the psychotropic effects of probiotics. Additionally, the immunoregulatory function of GABA participates in comorbidity mechanisms, and its deficiency leads to elevated pro-inflammatory cytokine levels in the intestinal mucosa of comorbid patients, forming a vicious cycle of inflammation-neurotransmitter dysregulation (Seifi et al., 2018).

#### 4.1.3 Dopamine and norepinephrine

Besides 5-HT and GABA, the catecholamine neurotransmitters dopamine and norepinephrine are important in comorbidity pathogenesis. Maini Rekdal et al. (2019) elucidated the molecular mechanisms by which gut bacteria metabolize the Parkinson disease drug levodopa (L-dopa). The study determined that from *Enterococcus faecalis* tyrosine decarboxylase can decarboxylate L-dopa to dopamine, and that *Eggerthella lenta* dopamine dehydroxylase can further convert dopamine to m-tyramine. This discovery was not only of significant clinical importance for Parkinson disease drug treatment but also provided new perspectives for understanding how gut microbiota influences host catecholamine metabolism.

Dopamine receptors are widely expressed in intestinal tissues and participate in regulating gut motility and secretory functions. D2-like receptor activation inhibits gut motility, while D1-like receptors promote peristalsis. Belujon and Grace (2017) systematically elaborated in their review on the central role of dopamine system dysregulation in MDD, noting that anhedonia, a core symptom of depression, is closely related to mesolimbic dopamine pathway dysfunction. Microbiota dysbiosis affects the tyrosine supply and microbial-derived dopamine production and may exacerbate central dopamine deficiency states.

Norepinephrine system dysregulation is involved in comorbidity pathogenesis mainly through stress response mechanisms. Norepinephrine regulates intestinal function through α and β adrenergic receptors, and β receptor activation inhibits gut motility and secretion, an important mechanism for worsening constipation symptoms under stress conditions (Foster et al., 2017). More importantly, Agus et al. (2018) review noted that IDO activation shunts tryptophan toward the kynurenine pathway under chronic inflammatory conditions, while inflammatory processes consume tyrosine for acute-phase protein synthesis, leading to the competitive depletion of several neurotransmitter precursors. This metabolic reprograming is a key molecular mechanism connecting intestinal inflammation with neuropsychiatric symptoms.

### 4.2 Immune-inflammatory mechanisms

Immune-inflammatory responses are another core mechanism linking gut microbiota dysbiosis to comorbidity. Dysbiosis-induced immune activation affects the intestinal microenvironment and transmits inflammatory signals to the CNS, causing neuroinflammation and dysfunction.

#### 4.2.1 Pro-inflammatory cytokines

Pro-inflammatory cytokines are key molecular bridges connecting gut dysbiosis, peripheral inflammation, and central dysfunction. Serum IL-6 is significantly elevated in depression patients compared with healthy controls (Osimo et al., 2020). In patients with chronic constipation–depression comorbidity, IL-6 levels are further elevated relative to those with constipation alone, indicating more severe immune activation in the comorbid state. IL-6 influences brain function by entering the CNS via circumventricular organs, activating cerebrovascular endothelial cells, and stimulating vagal afferent fibers (Miller and Raison, 2016). TNF-α damages ICC and enteric neurons, causing abnormal slow-wave activity and impaired intestinal contractions (Grubisic et al., 2020). Through the NF-κB signaling pathway, TNF-α also drives cascading cytokine production, activates CNS microglia, and impairs hippocampal neurogenesis and synaptic plasticity (Hodes et al., 2015). IL-1β is generated via NLRP3 inflammasome activation. It inhibits hippocampal neurogenesis, reduces BDNF expression, and activates the HPA axis, resulting in sustained cortisol elevation (Hodes et al., 2015; Fleshner et al., 2017). Elevated LPS from gram-negative bacterial overgrowth activates TLR4-mediated innate immunity, triggering further IL-1β release (Levy et al., 2017). The downstream consequences for intestinal barrier integrity and systemic inflammation are described in sections 4.2.2 and 4.2.3.

#### 4.2.2 Intestinal barrier dysfunction

Sustained cytokine production and intestinal barrier dysfunction reinforce each other in a vicious cycle. The intestinal barrier consists of the mucus layer, epithelial tight junctions, and gut-associated lymphoid tissue. In comorbid patients, expression of tight junction proteins—including occludin, ZO-1, and claudin-1—is significantly decreased (Parada Venegas et al., 2019; Margolis et al., 2021).

TNF-α and IL-1β activate MLCK, causing tight junction protein phosphorylation and cytoskeletal reorganization. Butyrate deficiency simultaneously deprives epithelial cells of their primary energy source, compounding barrier damage (Parada Venegas et al., 2019). Clinically, serum zonulin and plasma LPS are both significantly elevated in patients with anxiety or depression, and both correlate positively with depression severity (Stevens et al., 2018).

Psychological stress and CRH increase intestinal permeability through mast cell-dependent mechanisms (Vanuytsel et al., 2014). CRH activates mast cell degranulation, releasing histamine, tryptase, and TNF-α, which further disrupt barrier integrity. This establishes a self-reinforcing cycle: stress → HPA activation → barrier damage → microbial translocation → systemic inflammation → neuroinflammation → worsening depression → increased stress.

#### 4.2.3 Systemic inflammatory response

Intestinal barrier failure leads to systemic low-grade chronic inflammation, a key pathological bridge between gut and brain. CRP is generally elevated in depression patients, and this persistent inflammatory state exerts cumulative damage on the nervous system (Osimo et al., 2019).

Systemically, pro-inflammatory cytokines induce IDO expression, shifting tryptophan metabolism toward the kynurenine pathway. This increases the neurotoxic metabolite QUIN while reducing the neuroprotective metabolite KYNA (Savitz, 2020). Serum BDNF is reduced by approximately 30% in depression patients, compromising neuronal survival, synaptic plasticity, and hippocampal neurogenesis (Kishi et al., 2017). Oxidative stress and inflammation mutually reinforce one another, jointly damaging neurons, glial cells, and BBB integrity (Hodes et al., 2015).

### 4.3 The HPA axis

Immune-inflammatory mechanisms are closely intertwined with the neuroendocrine system, in which the HPA axis is the core regulatory system for the body’s response to stress, and its dysfunction is one of the most important biological markers of depression. Foster et al. (2017) review systematically elaborated on the regulatory mechanisms of the microbiome on stress responses and the HPA axis. Classic germ-free mouse experiments demonstrated that animals lacking gut microbiota exhibit significantly enhanced HPA axis reactivity, with corticosterone responses to stress that were 2–3-fold higher than conventional mice. More importantly, colonization with conventional microbiota during early life (within 14 days after birth) reversed this HPA axis hyperreactivity, but colonization in adulthood had limited effects, suggesting the existence of a critical developmental window, a finding of great guiding importance for early intervention strategies.

Juruena et al. (2018) systematic review detailed the potential of HPA axis function as a depression biomarker. Comorbid patients exhibit significant HPA axis dysfunction characteristics: elevated morning cortisol levels, flattened normal diurnal rhythm, and diminished response to the dexamethasone suppression test. Chronic hypercortisolism exposure has particularly significant damaging effects on the hippocampus, leading to neuronal dendritic atrophy, reduced synaptic density, and neurogenesis inhibition, resulting in an average 8–10% reduction in hippocampal volume in depression patients (Cryan et al., 2019).

Cortisol damages the CNS and has widespread effects on the gut. Karl et al. (2017) demonstrated that prolonged physiological stress simultaneously alters gut microbiota composition and increases intestinal permeability. Specific manifestations include the direct damaging effects of cortisol on the intestinal barrier and alteration of gut microbiota composition: Bacteroidetes phylum abundance increases, Firmicutes phylum abundance decreases, and beneficial bacteria such as *Bifidobacterium* and *Lactobacillus* are reduced. van de Wouw et al. (2018) reported that SCFAs, particularly butyrate, regulate glucocorticoid receptor (GR) expression through HDAC inhibition, affecting HPA axis negative feedback regulation efficiency. From a therapeutic perspective, Nikolova et al. (2021) meta-analysis confirmed that probiotic interventions significantly reduced salivary cortisol responses in healthy volunteers by approximately 25%, while improving anxiety and depression scores, providing evidence-based support for microbiota-targeted therapy.

### 4.4 SCFA pathways

As discussed across preceding sections, SCFAs are central mediators linking gut microbiota to intestinal barrier integrity, immune regulation, neurotransmitter biosynthesis, and neuroprotection. Acetate, propionate, and butyrate are the principal end products of microbial fermentation of dietary fiber (Koh et al., 2016; Dalile et al., 2019). In chronic constipation patients, the abundance of butyrate-producing bacteria such as *Faecalibacterium prausnitzii* is markedly reduced, and fecal butyrate concentration is correspondingly decreased—changes that likely contribute to constipation development (Dimidi et al., 2017). The role of butyrate as the primary colonocyte energy substrate is discussed in section 2.2.3.

As an HDAC inhibitor, butyrate upregulates tight junction proteins (occludin, claudins, ZO-1) and the mucin MUC2, reinforcing intestinal barrier function (Dalile et al., 2019). Butyrate also promotes colonic Treg differentiation by inducing Foxp3 expression (Furusawa et al., 2013), and suppresses NF-κB signaling to reduce pro-inflammatory cytokine production.

At the CNS level, butyrate crosses the BBB and upregulates BDNF expression, promoting neuroplasticity. It also inhibits pro-inflammatory microglial polarization and reduces neuroinflammation (Silva et al., 2020; Erny et al., 2015). Microbiome-derived SCFAs are essential for normal microglia maturation; germ-free mice exhibit microglia developmental defects, which SCFA supplementation can rescue (Erny et al., 2015). For gut motility, SCFAs activate GPR41/43 receptors on EC cells to stimulate 5-HT release and initiate peristaltic reflexes—a mechanism detailed in section 4.1.1 (Dalile et al., 2019). In STC patients, reduced total fecal SCFA concentration impairs colonic motility through converging mechanisms: epithelial energy deficit, diminished 5-HT release, and weakened barrier function (Dimidi et al., 2017).

### 4.5 Vagal nerve pathways

In the above molecular mechanisms, the vagus nerve is the main neural pathway between the gut and brain, performing key information transmission functions. Bonaz et al. (2018) review detailed the central position of the vagus nerve in the MGBA. The vagus nerve is the tenth cranial nerve, of which 80% are afferent fibers that transmit mechanical, chemical, and immune signals from the gut to the CNS. Microbial metabolites, 5-HT released by EC cells, and bacterial products transmit signals to the brainstem nucleus tractus solitarius (NTS) by activating various receptors on vagal nerve endings, and then project to the brain regions closely related to mood regulation, including the locus coeruleus, dorsal raphe nucleus, amygdala, and hippocampus.

The therapeutic potential of the vagus nerve has been validated clinically. Bottomley et al. (2019) systematic review and meta-analysis demonstrated that vagus nerve stimulation (VNS) is an effective method for treating treatment-resistant depression, with approximately 30–40% of patients exhibiting significant clinical responses. Depression patients generally exhibit reduced vagal tone and decreased heart rate variability (HRV), suggesting parasympathetic insufficiency. Bercik et al. (2011) demonstrated that the improving effects of probiotics on mood and gut symptoms depend on intact vagal nerve function, and vagotomy can abolish the anxiolytic effects of *Bifidobacterium longum* NCC3001.

Vagus nerve-mediated bidirectional communication creates dynamic interactions between mood and gut function (Bonaz et al., 2018; Margolis et al., 2021). Negative emotions such as anxiety and depression reduce vagal tone, weaken parasympathetic pro-motility effects on the gut, and reduce intestinal motility, while abdominal discomfort and psychological distress caused by constipation activate the sympathetic nervous system, further inhibiting intestinal motility and forming a vicious cycle. Margolis et al. (2021) emphasized that understanding this bidirectional regulatory mechanism is crucial for developing comprehensive treatment strategies that simultaneously improve mood and gastrointestinal symptoms.

### 4.6 Tryptophan metabolism

Tryptophan metabolism is a key metabolic network that integrates the above multiple pathways. As an essential amino acid, tryptophan is metabolized mainly through three pathways: the 5-HT pathway, the kynurenine pathway, and the microbial indole pathway. Cervenka et al. (2017) systematically elaborated on the multiple roles of kynurenine metabolites in exercise, inflammation, and mental health. Notably, approximately 95% of tryptophan is metabolized through the kynurenine pathway, with only 1–2% used for 5-HT synthesis. Pro-inflammatory cytokines, particularly IFN-γ, can induce IDO expression and activate the kynurenine pathway; depression patients have an elevated kynurenine/tryptophan ratio, reflecting significant metabolic redirection.

O’Mahony et al. (2015) detailed the relationship between tryptophan metabolism and the MGBA. When the kynurenine pathway is overactivated, tryptophan available for 5-HT synthesis is reduced, and this “tryptophan depletion” effect leads to a dual negative effect of low mood caused by central 5-HT deficiency and slowed intestinal motility caused by peripheral 5-HT insufficiency. Schwarcz et al. (2012) observed that the metabolic fate of kynurenine differs dramatically in different cell types: in microglia, it is metabolized to the neurotoxic metabolite QUIN, an NMDA receptor agonist that can lead to glutamate excitotoxicity; in astrocytes, it is metabolized to the neuroprotective metabolite KYNA.

Depression patients exhibit kynurenine pathway metabolic imbalance, manifested as a reduced KYNA/QUIN ratio, suggesting that neurotoxic metabolites predominate (Savitz, 2020). This metabolic imbalance works together with neuroinflammation and oxidative stress to exacerbate neuronal damage and dysfunction. From a therapeutic perspective, Nikolova et al. (2021) demonstrated that probiotic interventions can improve kynurenine metabolite balance through anti-inflammatory effects, providing a theoretical basis and practical pathways for improving tryptophan metabolism and improving neuropsychiatric symptoms through gut microbiota modulation. The major mechanistic pathways mediating chronic constipation-depression comorbidity via the MGBA are summarized in Table 3.

**TABLE 3:** Major mechanistic pathways mediating chronic constipation-depression comorbidity via the MGBA.

Mechanistic pathway (section)	Key biological mechanisms	Relevance to comorbidity pathogenesis	Key references
5-HT system (section 4.1.1)	∼95% of body 5-HT produced by intestinal EC cells; butyrate upregulates TPH1 expression via histone acetylation at TPH1 promoter; dysbiosis upregulates SERT and impairs EC cell function - > reduced colonic 5-HT; reduced 5-HT4 receptor activation (myenteric plexus) - > slowed peristalsis; overactivated 5-HT3 receptors - > visceral hypersensitivity; 5-HTTLPR short allele increases comorbidity susceptibility; > 60% of long-term SSRI users report constipation	5-HT deficiency simultaneously slows intestinal peristalsis and contributes to depressive symptoms; peripheral 5-HT cannot cross BBB directly but influences CNS indirectly via vagal activation, immune cell modulation, and tryptophan availability; antidepressant treatment may worsen gastrointestinal symptoms by altering peripheral 5-HT signaling	Yano et al., 2015; Yano et al., 2015; Dalile et al., 2019; Cryan et al., 2019; Loh et al., 2024; Socala et al., 2021; Cao et al., 2017; Agirman et al., 2021; Gershon, 2004)
GABA system (section 4.1.2)	Bacteroides spp. and Lactobacillus/Bifidobacterium produce GABA via glutamate decarboxylase (GAD); gut GABA acts via ionotropic GABA-A and metabotropic GABA-B receptors on enteric neurons; ∼30% of enteric neurons simultaneously express both 5-HT and GABA; prefrontal cortex GABA concentration decreases 18–22% in depression patients, positively correlated with gut GABA-producing microbial abundance	Gut-derived GABA modulates intestinal motility and stress-induced colonic inflammation; fecal Bacteroides abundance negatively correlated with functional connectivity in depression-related brain regions (r = -0.67, P = 0.0005); probiotic L. rhamnosus JB-1 increased GABA-B receptor expression in hippocampus/cortex and improved depression- and anxiety-like behaviors; vagotomy completely abolished these beneficial effects	(Strandwitz et al., 2019; Seifi et al. (2018); Bravo et al., 2011)
Dopamine and norepinephrine (section 4.1.3)	Enterococcus faecalis tyrosine decarboxylase converts L-dopa - > dopamine in gut; Eggerthella lenta converts dopamine - > m-tyramine; dopamine D2-like receptors inhibit gut motility; D1-like receptors promote peristalsis; beta-adrenergic receptor activation inhibits gut motility and secretion (stress-mediated constipation); IDO activation under chronic inflammation depletes tyrosine for acute-phase protein synthesis, reducing catecholamine precursor availability	Microbiota dysbiosis affects catecholamine precursor supply and microbial-derived dopamine production, potentially exacerbating central dopamine deficiency; anhedonia (core depression symptom) closely related to mesolimbic dopamine dysfunction; beta-adrenergic inhibition of gut motility is key mechanism for constipation worsening under stress conditions	(Belujon and Grace, 2017; Foster et al., 2017; Grubisic et al., 2020; Agus et al., 2018)
Pro-inflammatory cytokines (section 4.2.1)	Dysbiosis-induced LPS activates TLR4 - > NF-kappaB - > IL-6, TNF-alpha, IL-1beta (via NLRP3 inflammasome activation); IL-6: enters CNS via circumventricular organs, activates cerebrovascular endothelium, stimulates vagal afferents; TNF-alpha: damages ICC/enteric neurons, impairs slow-wave activity, activates CNS microglia, impairs hippocampal neurogenesis and synaptic plasticity; IL-1beta: inhibits hippocampal neurogenesis, reduces BDNF expression, activates HPA axis - > sustained cortisol elevation	Pro-inflammatory cytokines form central mechanistic bridge between gut dysbiosis and CNS dysfunction; IL-6 levels significantly elevated in depression vs. controls (meta-analysis); in comorbid patients, IL-6 levels further elevated relative to constipation alone, suggesting synergistic inflammatory activation; cytokine-driven ICC and enteric neuron damage directly links inflammation to gut dysmotility	(Hodes et al., 2015; Osimo et al. (2020); Hodes et al., 2015; Fleshner et al., 2017; Parada Venegas et al., 2019)
Intestinal barrier dysfunction (section 4.2.2)	TNF-alpha and IL-1beta activate MLCK - > tight junction protein phosphorylation and cytoskeletal reorganization; expression of occludin, ZO-1, claudin-1 significantly decreased in comorbid patients; butyrate deficiency deprives colonocytes of primary energy source, compounding barrier damage; psychological stress + CRH increase intestinal permeability via mast cell degranulation - > histamine, tryptase, TNF-alpha release	Increased intestinal permeability allows LPS/peptidoglycan translocation - > systemic endotoxemia (“leaky gut”); serum zonulin and plasma LPS significantly elevated in anxiety/depression patients, correlating positively with depression severity; self-reinforcing cycle: stress - > HPA activation - > barrier damage - > microbial translocation - > systemic inflammation - > neuroinflammation - > worsening depression - > increased stress	(Margolis et al., 2021; Osimo et al., 2019; Vanuytsel et al., 2014; Stevens et al., 2018)
HPA axis regulation (section 4.3)	Germ-free mice: 2–3x elevated corticosterone vs. conventional mice; colonization within 14 days post-birth reverses HPA hyperreactivity but adult colonization has limited effect (critical developmental window); comorbid patients: elevated morning cortisol, flattened diurnal rhythm, impaired dexamethasone suppression test response; chronic hypercortisolism - > ∼8–10% reduction in hippocampal volume (neuronal dendritic atrophy, reduced synaptic density, neurogenesis inhibition)	Cortisol inhibits gut motility, reduces intestinal blood flow, impairs gut barrier integrity, alters microbiota (increases Bacteroidetes, decreases Bifidobacterium/Lactobacillus); SCFAs (butyrate) regulate glucocorticoid receptor (GR) expression via HDAC inhibition, modulating HPA negative feedback efficiency; probiotic interventions reduced salivary cortisol responses by ∼25% while improving anxiety and depression scores (meta-analysis, *n* = 7 RCTs, 404 patients)	(Sudo et al., 2004; Grubisic et al., 2020; Karl et al., 2017; van de Wouw et al., 2018; Nikolova et al., 2021; Dimidi et al., 2017)
SCFA signaling (section 4.4)	Acetate, propionate, butyrate produced by microbial fermentation of dietary fiber; butyrate: ∼70% of colonocyte energy requirements; HDAC inhibitor - > upregulates occludin, claudins, ZO-1, MUC2 (barrier reinforcement); promotes colonic Treg differentiation via Foxp3 induction; suppresses NF-kappaB to reduce pro-inflammatory cytokines; crosses BBB - > upregulates BDNF, inhibits pro-inflammatory microglial polarization, essential for normal microglia maturation (germ-free mice have microglia defects rescued by SCFA supplementation); activates GPR41/43 on EC cells to stimulate 5-HT release and initiate peristaltic reflexes	Butyrate-producing bacteria (Faecalibacterium, Roseburia, Coprococcus) markedly reduced in both constipation and depression; STC patients: reduced total fecal SCFA impairs colonic motility via converging mechanisms (epithelial energy deficit + diminished 5-HT release + weakened barrier); SCFA deficiency links gut dysbiosis to both constipation pathophysiology and neuropsychiatric dysfunction simultaneously	(Koh et al., 2016; Dalile et al., 2019; Bottomley et al., 2019; Erny et al., 2015; Silva et al., 2020; Furusawa et al., 2013)
Vagal nerve pathways (section 4.5)	80% afferent vagal fibers transmit microbial metabolites, EC cell 5-HT, and bacterial products - > NTS - > locus coeruleus, dorsal raphe nucleus, amygdala, hippocampus; depression patients show reduced vagal tone and decreased heart rate variability (HRV), indicating parasympathetic insufficiency; VNS effective in ∼30–40% of treatment-resistant depression cases	Vagal pathway mediates bidirectional gut-brain communication; probiotic anxiolytic effects (Bifidobacterium longum NCC3001) depend on intact vagal function—vagotomy abolishes effects; negative emotions reduce vagal tone, weakening parasympathetic pro-motility effects on gut - > constipation; abdominal discomfort from constipation activates sympathetic NS, further inhibiting intestinal motility—vicious cycle; understanding this pathway is crucial for comprehensive treatment strategies	(Bonaz et al., 2018; Bercik et al., 2011; Cervenka et al., 2017; Stevens et al., 2018)
Tryptophan metabolism (section 4.6)	∼95% of Trp metabolized via kynurenine pathway; IDO/TDO activated by IFN-gamma and pro-inflammatory cytokines; in microglia: kynurenine - > QUIN (neurotoxic NMDA receptor agonist - > glutamate excitotoxicity, neuronal damage); in astrocytes: kynurenine - > KYNA (neuroprotective); depression patients: elevated Kyn/Trp ratio, reduced KYNA/QUIN ratio (neurotoxic metabolites predominate); comorbid patients show elevated plasma kynurenine/tryptophan ratios positively correlated with both constipation severity and depression scores	Kynurenine overactivation depletes Trp for 5-HT synthesis - > dual negative effect: central 5-HT deficiency (low mood) + peripheral 5-HT insufficiency (slowed intestinal motility); metabolic imbalance works together with neuroinflammation and oxidative stress to exacerbate neuronal damage; probiotic interventions improve kynurenine metabolite balance via anti-inflammatory effects; tryptophan depletion provides a unified mechanistic explanation for both depression and constipation simultaneously	(Agus et al., 2018; O’Mahony et al., 2015; Schwarcz et al., 2012; Parthasarathy et al., 2016; Myint and Kim, 2014; Dimidi et al., 2017)

5-HT, serotonin; EC, enterochromaffin; TPH1, tryptophan hydroxylase 1; SERT, serotonin reuptake transporter; GABA, gamma-aminobutyric acid; GAD, glutamate decarboxylase; HPA, hypothalamic-pituitary-adrenal; SCFAs, short-chain fatty acids; HDAC, histone deacetylase; BDNF, brain-derived neurotrophic factor; LPS, lipopolysaccharide; TLR4, Toll-like receptor 4; NF-kappaB, nuclear factor kappa B; MLCK, myosin light-chain kinase; VNS, vagus nerve stimulation; HRV, heart rate variability; ICC, interstitial cells of Cajal; IDO, indoleamine 2,3-dioxygenase; Trp, tryptophan; Kyn, kynurenine; QUIN, quinolinic acid; KYNA, kynurenic acid; NMDA, N-methyl-D-aspartate. The mechanistic pathways described are largely inferred from preclinical models, in vitro experiments, and observational human studies. Direct causal evidence in human comorbid populations remains limited. All pathways should be interpreted as plausible, evidence-informed hypotheses rather than established mechanisms.

### 5 Specific alterations in gut microbiota composition

Gut microbiota composition alterations represent an important pathological feature of the comorbidity between chronic constipation and depression. Recent high-throughput sequencing technological advances have enabled researchers to systematically characterize the microbial profiles associated with both conditions and their comorbid states. A comprehensive understanding of these specific alterations is essential for elucidating comorbidity mechanisms, developing diagnostic biomarkers, and formulating precision therapeutic strategies. Figure 2 presents these specific gut microbiota alterations.

### 5.1 Gut microbiota characteristics in constipated patients

#### 5.1.1 Changes in dominant bacterial taxa

The gut microbiota composition in patients with chronic constipation exhibits significant and consistent alteration patterns. Parthasarathy et al. (2016) systematically analyzed colonic mucosal and fecal microbiota in 25 female patients with chronic constipation and 25 healthy controls, and demonstrated that colonic mucosal microbiota could distinguish constipated patients from healthy controls with 94% accuracy, independent of colonic transit time. At the phylum level, Bacteroidetes was more abundant in the colonic mucosa of the constipated patients, whereas fecal microbiota composition was associated with colonic transit time. Within the Firmicutes phylum, *Faecalibacterium*, *Lactococcus, and Roseburia* positively correlated with faster colonic transit time. Dimidi et al. (2017) reported that patients with functional constipation exhibited significantly reduced fecal counts of *Bifidobacterium* and *Lactobacillus*.

At the genus and species levels, a significant reduction in key SCFA-producing bacteria represents a core feature of constipated patients. *F. prausnitzii*, the most important butyrate-producing bacterium, demonstrated significantly decreased abundance in constipated patients (Lopez-Siles et al., 2017). Lopez-Siles et al. (2017) indicated that *F. prausnitzii* constitutes approximately 5% of healthy adult gut microbiota, and that its abundance is reduced in various intestinal disorders. Zhuang et al. (2019) demonstrated that constipated patients exhibited significantly reduced abundances of *Faecalibacterium*, Ruminococcaceae, and *Roseburia*, decreased SCFA production, and elevated iso-butyrate levels. Furthermore, this study identified Desulfovibrionaceae as an important endotoxin producer enriched in constipated patients; its metabolic product hydrogen sulfide (H2S) inhibits colonic motility and promotes inflammation.

Proteobacteria, particularly Enterobacteriaceae, are significantly elevated in constipated patients and closely associated with intestinal inflammation and barrier dysfunction (Shin et al., 2015). Shin et al. (2015) defined Proteobacteria as a “microbial signature” of gut microbiota dysbiosis.

Different constipation types exhibit distinct microbiota characteristics. Patients with STC have increased methanogenic archaea, as methane can slow intestinal peristalsis and prolong transit time (Kunkel et al., 2011). Kunkel et al. (2011) systematic review and meta-analysis confirmed the significant association between breath methane positivity and constipation. Tap et al. (2017) identified unique microbiota signatures in patients with constipation-predominant IBS (IBS-C), including specific microbial composition alterations and reduced microbiota stability.

#### 5.1.2 Reduced diversity

Gut microbial diversity is an important indicator for assessing intestinal ecosystem health. Vandeputte et al. (2016) demonstrated in a large-scale cohort study that stool consistency was strongly associated with gut microbiota richness and composition, with hard stools (an indicator of constipation) correlating with lower microbial richness. β-Diversity analysis revealed significant differences in microbiota composition between constipated patients and healthy individuals (Parthasarathy et al., 2016).

Reduced microbial diversity exerts multifaceted negative effects on intestinal function. Roager et al. (2016) demonstrated that colonic transit time is related to bacterial metabolism and mucosal turnover, with prolonged colonic transit time associated with increased protein fermentation and decreased carbohydrate fermentation. Lozupone et al. (2012) indicated that low-diversity microbial communities have reduced functional redundancy and are more susceptible to external factors such as antibiotics, dietary changes, and stress.

#### 5.1.3 Characteristic biomarker bacteria

Through linear discriminant analysis effect size (LEfSe) and machine learning approaches, several characteristic biomarker bacteria have been identified for chronic constipation. Tap et al. (2017) demonstrated that microbiota composition significantly correlated with symptom severity.

Among depleted beneficial bacteria, *F. prausnitzii* represents the most important constipation-associated biomarker (Lopez-Siles et al., 2017). Butyrate produced by this bacterium provides energy for colonic epithelial cells and regulates gene expression by inhibiting DAC, exerting anti-inflammatory and barrier-protective functions. *Bifidobacterium* is significantly reduced, where these strains produce acetate and lactate, lower intestinal pH, and inhibit the growth of harmful bacteria (Dimidi et al., 2017).

Among enriched potentially harmful bacteria, Parker et al. (2020) systematically analyzed the relationship of *Alistipes* with inflammation, cancer, and mental health, noting that the functional role of this genus is complex and context-dependent.

### 5.2 Gut microbiota characteristics in patients with depression

#### 5.2.1 Reduced butyrate-producing bacteria

Gut microbiota alterations in patients with depression share some similarities with those in constipated patients, but also exhibit important differences. Valles-Colomer et al. (2019) systematically analyzed the neuroactive potential of human gut microbiota and its relationship with quality of life and depression in the Flemish Gut Flora Project cohort (*n* = 1,054), with validation in an independent dataset (*n* = 1,070). The study determined that butyrate-producing *Faecalibacterium* and *Coprococcus* were consistently correlated with higher quality of life indicators, while *Coprococcus* and *Dialister* were significantly reduced in patients with depression, even after adjusting for the effects of antidepressant medication.

Zheng et al. (2016) demonstrated through FMT experiments that gut microbiota composition in patients with MDD differed significantly from those in healthy controls, characterized by altered relative abundances of Firmicutes, Actinobacteria, and Bacteroidetes. Transplantation of “depression microbiota” from MDD patients into germ-free mice induced depressive-like behaviors, demonstrating that microbiota dysbiosis may play a causal role in the development of depressive-like behaviors.

Radjabzadeh et al. (2022) analyzed the Rotterdam cohort (*n* = 1,054) with validation in the Amsterdam HELIUS cohort (*n* = 1,539), identifying 13 microbial taxa associated with depressive symptoms, including Eggerthella, Subdoligranulum, Coprococcus, Sellimonas, Lachnoclostridium, Hungatella, and Ruminococcaceae family members.

#### 5.2.2 Alterations in GABA- and 5-HT-producing bacteria

Alterations in neurotransmitter-producing bacterial communities represent one of the most functionally significant features of gut microbiota in patients with depression. Strandwitz et al. (2019) systematically identified GABA-producing bacteria in the human gut, reporting that multiple *Bacteroides* species produce substantial amounts of GABA. Through analysis of fecal samples and functional magnetic resonance imaging data from 23 MDD patients, the study determined that the relative abundance of fecal *Bacteroides* was significantly negatively correlated with functional connectivity in depression-related brain regions (*r* = −0.67, *P* = 0.0005).

Regarding 5-HT-related bacteria, Yano et al. (2015) demonstrated that spore-forming bacteria (primarily Clostridia) promote 5-HT biosynthesis by colonic EC cells, supplying 5-HT to the colonic mucosa, lumen, and circulating platelets. Germ-free mice exhibited significantly reduced 5-HT levels in the colon and serum, which were restored following colonization with spore-forming bacteria. Furthermore, the study identified specific microbial metabolites (such as deoxycholate) that stimulate EC cells to upregulate TPH1 expression and promote 5-HT synthesis. Roager and Licht (2018) systematically analyzed microbial tryptophan catabolites in health and disease.

#### 5.2.3 Increased pro-inflammatory bacteria

The “inflammation hypothesis” of depression has gained widespread support, with gut microbiota dysbiosis being an important factor in initiating and maintaining low-grade chronic inflammation (Miller and Raison, 2016). Miller and Raison (2016) systematically elaborated on the role of inflammation in depression. Stevens et al. (2018) enrolled 50 participants (22 patients with anxiety/depression and 28 healthy controls) and determined that patients with anxiety or depression exhibited significantly elevated plasma levels of LPS, zonulin, and fatty acid-binding protein 2 (FABP2), indicating intestinal barrier dysfunction and bacterial product translocation.

Caso et al. (2021) recruited 46 patients with active MDD, 22 patients in remission, and 45 healthy controls, finding that the MDD patients exhibited elevated abundances of *Bilophila* (2-fold) and *Alistipes* (1.5-fold), while abundances of *Anaerostipes* (1.5-fold) and *Dialister* (15-fold) were significantly reduced. LPS levels in patients with active MDD were significantly higher than those in the controls and patients in remission.

Smith et al. (2013) demonstrated that SCFAs regulate the size and function of the colonic Treg pool through a free fatty acid receptor 2 (Ffar2/GPR43)-dependent mechanism, revealing the fundamental role of microbial metabolites in the co-adaptation of adaptive immunity and microbiota.

### 5.3 Gut microbiota characteristics in comorbid states

#### 5.3.1 Synergistic alterations

It must be explicitly acknowledged that the “synergistic alterations” described in this section are largely inferred by overlaying independent findings from constipation-only and depression-only cohorts. To our knowledge, no large-scale microbiome profiling study has directly compared comorbid patients (meeting diagnostic criteria for both conditions simultaneously) against single-disease and healthy control groups within the same dataset. This represents a critical gap in the field. The comorbid microbiota signatures described herein should therefore be interpreted as hypothesis-generating extrapolations rather than established findings, and dedicated cohort studies with rigorous comorbidity ascertainment are urgently needed.

Gut microbiota dysbiosis in patients with comorbid chronic constipation and depression may exhibit synergistic effects. Simpson et al. (2021) analyzed 26 studies in their systematic review and determined that anxiety and depression may be characterized by elevated abundances of pro-inflammatory bacteria (e.g., Enterobacteriaceae and *Desulfovibrio*) and reduced abundances of SCFA-producing bacteria (e.g., *Faecalibacterium*).

Both constipated patients and depressed patients exhibit reduced SCFA-producing bacteria such as *Faecalibacterium*, *Roseburia*, and *Coprococcus* (Valles-Colomer et al., 2019; Zhuang et al., 2019). Margolis et al. (2021) systematically elaborated on the role of the MGBA in intestinal motility and mood regulation, indicating that gut microbiota communicate bidirectionally with the CNS through neural, endocrine, and immune signaling pathways.

Reduced α-diversity is a common feature of both constipation and depression (Simpson et al., 2021; Vandeputte et al., 2016). This reduction in diversity may be more severe in comorbid states; however, original research data directly comparing comorbid patients with patients having single conditions are currently lacking.

#### 5.3.2 Inflammation-associated microbiota changes

Hall et al. (2017) performed longitudinal metagenomic sequencing on 20 IBD patients and 12 controls (266 total samples), determining that *Ruminococcus gnavus* was significantly enriched in the IBD patients, particularly during transient blooms coinciding with increased disease activity. The study identified two distinct *R. gnavus* strain clades, with the IBD-enriched clade containing 199 specific genes involved in oxidative stress responses, adhesion, iron acquisition, and mucin utilization. Lloyd-Price et al. (2019) comprehensively characterized gut microbial functional dysbiosis by integrating 1-year longitudinal follow-up data from 132 IBD patients.

#### 5.3.3 Correlation with disease severity

Gut microbiota characteristics demonstrate significant correlations with disease severity. Valles-Colomer et al. (2019) determined that *Faecalibacterium* and *Coprococcus* consistently correlated with higher quality of life. Zhuang et al. (2019) demonstrated that increasing butyrate concentrations through dietary fiber supplementation accelerated gastrointestinal transit and improved constipation symptoms.

Probiotic intervention studies have provided important evidence for the causal relationship between microbiota and disease. Nikolova et al. (2021) conducted a meta-analysis that included seven randomized controlled trials with 404 patients, and determined that probiotics as adjunctive therapy to antidepressants significantly improved depressive symptoms (standardized mean difference [SMD] = 0.83, 95% CI: 0.49–1.17), but were not effective as stand-alone treatment (SMD = −0.02). The gut microbiota studies in constipation, depression, and comorbid/overlapping conditions are summarized in Table 4.

**TABLE 4:** Gut microbiota studies in constipation, depression, and comorbid/overlapping conditions.

Reference	Condition	Population/ Sample	Sequencing/ method	Key microbial alterations	Primary condition	Functional implications for comorbidity
Lopez-Siles et al. (2017)	CC	25 female CC patients vs. 25 HC	16S rRNA (mucosal + fecal)	Mucosal: ↑ Bacteroidetes; ↓ Faecalibacterium, Lactococcus, Roseburia (positively correlated with faster transit); mucosal microbiota discriminated CC from HC with 94% accuracy; fecal microbiota composition associated with colonic transit time	Constipation	Mucosal microbiota may be more informative than fecal samples; SCFA-producing bacteria linked to transit time; microbiota-based discrimination feasible but limited to female STC population
Furusawa et al. (2013)	CC	Functional constipation patients (review)	Culture-based + 16S rRNA (review)	↓ Bifidobacterium; ↓ Lactobacillus; reduced fecal butyrate concentration; altered overall SCFA profiles	Constipation	Reduced SCFA-producing bacteria central to functional constipation; butyrate deficiency simultaneously impairs colonocyte energy supply, 5-HT release, and barrier integrity—convergent mechanisms for STC pathophysiology
Shin et al. (2015)	CC	Constipated patients + healthy controls	16S rRNA sequencing + metabolomics	↓ Faecalibacterium; ↓ Ruminococcaceae; ↓ Roseburia; ↓ total SCFAs; ↑ iso-butyrate; ↑ Desulfovibrionaceae (endotoxin/H2S producer); fiber supplementation increased butyrate and accelerated gastrointestinal transit	Constipation	H2S from Desulfovibrionaceae inhibits colonic motility and promotes inflammation; combined metabolomic-microbiota analysis reveals functional consequences; dietary fiber intervention provides proof of concept for SCFA-mediated transit improvement
Tap et al. (2017)	STC (archaea)	Systematic review + meta-analysis of STC studies	Breath methane testing (lactulose breath test)	Significantly elevated methane-positive breath tests in constipated vs. non-constipated patients; methanogenic archaea enriched in STC; methane slows intestinal peristalsis and prolongs transit time	Constipation (STC)	Methanogenic archaea represent an underappreciated microbial contributor to STC; may explain subtype-specific differences in depression comorbidity (STC > OOC)
Valles-Colomer et al. (2019)	Depression	1,054 (Flemish Gut Flora Project) + 1,070 (validation cohort)	16S rRNA sequencing + quality-of-life instruments	↓ Coprococcus; ↓ Dialister (persistent even after adjusting for antidepressant medication use); ↑ Faecalibacterium and Coprococcus correlated with higher quality of life indicators; consistent across two independent cohorts	Depression	First large-scale population study linking neuroactive microbiota potential to depression; antidepressant-controlled design strengthens findings; Coprococcus and Dialister as reproducible candidate biomarkers; highlights SCFA-producing taxa as correlates of mental well-being
Radjabzadeh et al. (2022)	Depression	MDD patients vs. HC; germ-free rat FMT recipients	16S rRNA + FMT experiment + behavioral testing	MDD patients: altered relative abundances of Firmicutes, Actinobacteria, Bacteroidetes vs. HC; transplantation of “depression microbiota” into germ-free mice induced depressive-like behaviors (anhedonia, anxiety-like) and altered tryptophan metabolism; healthy donor FMT reversed behavioral phenotypes	Depression	Preclinical causal evidence that MDD-associated microbiota can transfer depressive phenotype; tryptophan metabolism as key mechanistic mediator; FMT experimental design provides strongest available evidence for microbiota causality
Strandwitz et al. (2019)	Depression	23 MDD patients (fecal samples + fMRI)	16S rRNA + functional MRI (fMRI)	*Bacteroides* spp. produce substantial GABA in human gut; fecal Bacteroides abundance negatively correlated with functional connectivity in depression-related brain regions (*r* = −0.67, *P* = 0.0005)	Depression	Direct link between gut GABA-producing microbiota and depression-associated brain activity—functional (not merely taxonomic) mechanistic evidence; small sample limits generalizability but correlation effect size is large
Roager and Licht (2018)	Depression	1,054 (Rotterdam cohort) + 1,539 (Amsterdam HELIUS cohort)	16S rRNA sequencing	13 microbial taxa associated with depressive symptoms: ↑ Eggerthella; ↓ Subdoligranulum, Coprococcus, Sellimonas, Lachnoclostridium, Hungatella, Ruminococcaceae members; multi-cohort replication across two large independent datasets	Depression	Largest multi-cohort replication study of depression-microbiota associations; highlights both SCFA-producing and neuroactive taxa; Coprococcus depletion consistent with Valles-Colomer et al. findings, strengthening biomarker candidacy
Smith et al. (2013)	MDD (active vs. remission)	46 active MDD + 22 remission + 45 HC	16S rRNA + LPS measurement	Active MDD: ↑ Bilophila (2x); ↑ Alistipes (1.5x); ↓ Anaerostipes (1.5x); ↓ Dialister (15x reduction); LPS significantly elevated in active MDD vs. remission and HC	Depression (disease state)	Disease-state-dependent microbiota changes; LPS elevation confirms gut barrier failure and systemic endotoxemia in active MDD; Dialister depletion highly consistent across multiple independent cohorts; remission state associated with partial microbiota normalization
Simpson et al. (2021)	Anxiety/ Depression (systematic review)	26 studies, mixed populations	Systematic review (16S rRNA studies)	Consistent across studies: ↑ Enterobacteriaceae; ↑ Desulfovibrio; ↓ Faecalibacterium; ↓ SCFA-producing bacteria in anxiety and depression	Comorbidity (inferred)	Pro-inflammatory taxa enriched while SCFA producers consistently depleted in both conditions; supports shared dysbiosis signature hypothesis; however, direct comorbid cohort data lacking—findings represent hypothesis-generating extrapolations
Yano et al. (2015)	Constipation + Depression shared 5-HT pathway	Germ-free mice + conventional mice + colonization experiments	Microbial colonization + HPLC 5-HT measurement + *ex vivo* experiments	Spore-forming bacteria (Clostridia) promote colonic EC cell 5-HT biosynthesis; germ-free mice: severely reduced colonic and serum 5-HT; restored upon colonization with spore-forming bacteria; deoxycholate (microbial metabolite) upregulates TPH1 expression; spore-forming bacteria regulate 5-HT supply to colonic mucosa, lumen, and circulating platelets	Shared mechanism (5-HT pathway)	Microbiota-derived signals drive peripheral 5-HT synthesis, linking gut microbial composition to both intestinal motility and mood regulation simultaneously; establishes mechanistic basis for how a single microbial community shift can contribute to both constipation and depression concurrently

CC, chronic constipation; STC, slow transit constipation; MDD, major depressive disorder; HC, healthy controls; 16S rRNA, 16S ribosomal RNA amplicon sequencing; FMT, fecal microbiota transplantation; fMRI, functional magnetic resonance imaging; SCFAs, short-chain fatty acids; LPS, lipopolysaccharide; 5-HT, serotonin; GABA, gamma-aminobutyric acid. Arrows: up-arrow = increased relative abundance vs. healthy controls; down-arrow = decreased relative abundance vs. healthy controls. The “Comorbidity (inferred)” designation indicates that findings are extrapolated by overlapping results from independent single-disease cohorts. To our knowledge, no large-scale microbiome profiling study has directly compared comorbid patients (meeting diagnostic criteria for both constipation and depression simultaneously) against single-disease and healthy control groups within the same dataset. Comorbid microbiota signatures should be interpreted as hypothesis-generating extrapolations; dedicated cohort studies are urgently needed.

## 6 Intervention strategies

### 6.1 Probiotics and prebiotics

#### 6.1.1 The concept of psychobiotics

Psychobiotics are defined as live microorganisms that confer mental health benefits to the host when ingested in adequate amounts ( Śliwka et al., 2025). Psychobiotics have recently garnered considerable attention as novel interventions for modulating the gut-brain axis (Cui et al., 2025). Psychobiotics exert their effects through several mechanisms, including vagus nerve activation, enteroendocrine cell stimulation, immune signaling modulation, and neuroactive metabolite production (Zielinska et al., 2022). A systematic review that analyzed 45 studies published between 2020 and 2024 revealed that *Lactobacillus* and *Bifidobacterium* are the most extensively studied psychobiotic genera, accounting for 45.5 and 29% of the total studies, respectively ( Śliwka et al., 2025).

#### 6.1.2 Selection of specific strains

Different bacterial strains exhibit distinct neuromodulatory properties. Strandwitz et al. demonstrated that *Bacteroides* species produce substantial quantities of GABA, and that the relative abundance of fecal *Bacteroides* negatively correlates with functional connectivity in brain regions exhibiting elevated activity during depression in patients with depressive disorders (Strandwitz et al., 2019). *Lactobacillus* and *Bifidobacterium* are considered the genera with the greatest psychobiotic potential, capable of producing neuroactive substances that act through the gut-brain axis (Zielinska et al., 2022). Clinical research confirmed that a 30-day combined administration of *Lactobacillus helveticus* R0052 and *B. longum* R0175 significantly reduced Hospital Anxiety and Depression Scale (HADS) global scores and Hopkins Symptom Checklist (HSCL-90) global severity indices in healthy volunteers, alleviating somatization, depression, and anger-hostility symptoms (Messaoudi et al., 2011). A systematic review and meta-analysis of psychobiotic interventions for anxiety disorders in adolescents demonstrated that psychobiotic interventions significantly reduced anxiety levels (Cohen Kadosh et al., 2021).

#### 6.1.3 Synergistic effects of prebiotics

Prebiotics are substrates that are selectively fermented to promote the growth and activity of beneficial gut microorganisms (Gibson et al., 2017). Prebiotics enhance SCFA production, particularly butyrate, propionate, and acetate (Dalile et al., 2019). These metabolites can cross the BBB, modulate neuroinflammation by inhibiting HDAC activity, and enhance barrier integrity (Berding et al., 2021). SCFAs are important neural mediators that may directly influence BBB integrity, neurotransmission, neurotrophic factors, and 5-HT biosynthesis (Silva et al., 2020).

### 6.1.4 Clinical evidence

Recent meta-analyses have demonstrated that probiotic, prebiotic, or synbiotic interventions significantly improve symptoms of depression, anxiety, and stress (Amirani et al., 2020). Multiple systematic reviews have confirmed that probiotic and prebiotic interventions significantly alleviate depressive and anxiety symptoms, with *Bifidobacterium* and *Lactobacillus* strains demonstrating the most consistent beneficial effects ( Śliwka et al., 2025; Amirani et al., 2020). However, considerable inter-individual variability in psychobiotic efficacy have been noted, which may be attributed to baseline gut microbiota composition, genetic variations, and environmental factors (Berding et al., 2021).

### 6.2 Fecal microbiota transplantation

#### 6.2.1 Mechanisms of action

FMT transfers gut microbiota from a healthy donor to the recipient’s gastrointestinal tract to restore the intestinal ecological balance (Chinna Meyyappan et al., 2020). Preclinical studies have demonstrated that transplanting fecal microbiota from patients with MDD to rodents induced depression-like behaviors, including anhedonia and anxiety-like behaviors, and altered tryptophan metabolism, while microbiota transfer from healthy donors reversed these behavioral phenotypes (Kelly et al., 2016; Zhang et al., 2024). FMT exerts antidepressant effects through multiple mechanisms, including neurotransmitter level modulation, inflammatory marker reduction, gut barrier function improvement, and HPA axis activity regulation (Zhang et al., 2024).

#### 6.2.2 Current clinical applications

Systematic reviews have indicated that all included studies determined that transplantation from healthy microbiota reduces depression- and anxiety-like symptoms and behaviors (Chinna Meyyappan et al., 2020). A 2025 meta-analysis by Zhang et al. evaluated the efficacy of FMT in alleviating depressive symptoms, and demonstrated that the FMT group exhibited significantly reduced depressive symptoms compared to the control group (SMD = −1.21; *P* = 0.0003) (Zhang et al., 2025). This review cited Kurokawa et al.’s results that indicated that patients with Hamilton Depression Rating Scale (HAM-D) scores ≥ 8 exhibited reduced microbial diversity compared to healthy donors, and that increased microbial diversity following FMT treatment positively correlated with symptom improvement (Zhang et al., 2025). Clinical case reports have demonstrated that two MDD patients who received FMT as adjunctive therapy demonstrated improvement in depressive symptoms within 4 weeks, with effects persisting for 8 weeks in one patient (Doll et al., 2022).

### 6.2.3 Safety and efficacy assessment

Safety assessment of FMT remains an important consideration for clinical application. A study involving patients with IBD determined that the severity of depression, anxiety, and obsessive-compulsive symptoms significantly decreased following FMT (Bonam et al., 2020). However, long-term safety concerns, particularly the potential unintended consequences of targeted microbial manipulation, require further investigation (Berding et al., 2021). Recent meta-analyses have emphasized the need for larger-scale, rigorously designed randomized controlled trials to comprehensively determine FMT treatment efficacy and safety, while recommending that future research focus on establishing standardized analytical protocols and validating predictive biomarkers (Zhang et al., 2025).

### 6.3 Dietary interventions

#### 6.3.1 Mediterranean diet pattern

The Mediterranean diet (MD), characterized by high intake of plant-based foods, monounsaturated fats, and polyphenols (primarily from extra virgin olive oil), has been demonstrated to modulate gut microbiota composition and reduce the risk of metabolic, cardiovascular, and neurodegenerative diseases (Perrone and D’Angelo, 2025). A 2024 systematic review encompassing 37 studies (17 observational and 20 interventional) revealed that MD adherence correlates with increased microbial diversity, with *Faecalibacterium* and *Prevotella* being the bacterial genera most frequently demonstrating increased abundances in both observational and interventional studies (Khavandegar et al., 2024). The MD promotes the growth of beneficial gut bacteria, including *Bifidobacterium*, *F. prausnitzii*, and *Roseburia*, which produce SCFAs that enhance gut barrier integrity, reduce inflammation, and improve metabolic homeostasis (Perrone and D’Angelo, 2025).

Clinical studies have demonstrated that the MD is associated with improved cognitive function and reduced inflammatory markers such as CRP (Perrone and D’Angelo, 2025). Individuals who consumed more whole grains and vegetables exhibited significantly lower incidences of depression and anxiety (Firth et al., 2020). The anti-inflammatory effects of the MD are mediated through the gut-brain axis, with improvements in gut microbiota composition and increased SCFA concentrations being pivotal in this process (Barber et al., 2023).

#### 6.3.2 High-fiber diet

Dietary fiber is the primary substrate for gut microbial fermentation to produce SCFAs. High-fiber diets significantly increase gut microbial diversity and promote the growth of SCFA-producing bacteria such as *R. bromii* and *Eubacterium rectale* (Baxter et al., 2019). Dietary fiber intake negatively correlates with depressive symptom severity (Firth et al., 2020). Butyrate produced from fiber fermentation is essential for maintaining gut barrier function, serving as the primary energy source for colonic epithelial cells while demonstrating neuroprotective properties (Chen et al., 2021).

#### 6.3.3 Role of fermented foods

Fermented foods are rich in probiotics and bioactive compounds that can directly influence gut microbiota composition. Regular consumption of fermented foods is associated with increased gut microbial diversity and reduced inflammatory markers (Perrone and D’Angelo, 2025). However, the direct effects of fermented foods on mental health require confirmation through additional high-quality clinical studies (Marco et al., 2017).

#### 6.3.4 Personalized nutrition approaches

Personalized nutritional interventions based on individual gut microbiota composition are emerging as a research focus. Baseline gut microbiota is considered a primary factor influencing individual responses to dietary interventions (Barber et al., 2023). Future research should comprehensively map the interplay between microbial metabolic pathways, host genetic variations, and environmental factors to develop optimized personalized treatment regimens for different populations (Berding et al., 2021).

### 6.4 Novel perspectives in pharmacotherapy

#### 6.4.1 Effects of conventional medications on microbiota

Antidepressant medications have antimicrobial properties that can directly affect gut microbiota (Ait Chait et al., 2020). Ait Chait et al. demonstrated that multiple antidepressants exhibit significant dose-dependent inhibitory effects on human gut commensal strains, with desipramine (a tricyclic antidepressant) displaying the strongest antimicrobial activity, with minimum inhibitory concentrations of 75 and 150 μg/mL against *Akkermansia muciniphila* and *Escherichia coli*, respectively (Ait Chait et al., 2020). A systematic review and meta-analysis indicated that antidepressant intake in animal models of depression was linked to a distinct gut microbial profile as based on β-diversity analysis; however, this result was not observed in human studies. Regarding microbial composition, the genera *Faecalibacterium* and *Parasutterella* in antidepressant-treated patients with MDD were consistently depleted, along with *Bifidobacterium* enrichment (Gamboa et al., 2025).

#### 6.4.2 Novel microbiota-targeted therapeutics

The development of novel therapeutics based on gut microbial mechanisms is emerging. Lukić et al. discovered that antidepressants affect gut microbiota, and that *R. flavefaciens* can abolish the effects of antidepressants on depression-like behavior, indicating that gut microbiota plays an important modulatory role in antidepressant efficacy (Lukić et al., 2019). Fluoxetine induces increases in Erysipelotrichia and Proteobacteria, partially ameliorating chronic unpredictable mild stress (CUMS)-induced low bacterial diversity and alterations in microbial composition (Lukić et al., 2019).

#### 6.4.3 Combination therapy strategies

Clinical trials have demonstrated that combined treatment with probiotics and antidepressants is more effective than monotherapy (Ng et al., 2018). Jiang et al. confirmed that at the genus level, *Ruminococcus*, *Bifidobacterium*, *Subdoligranulum*, *Faecalibacterium*, and *Blautia* were more abundant in selective SSRI responders, while *Dialister*, *Streptococcus*, and *Escherichia-Shigella* were more abundant in non-responders. These differential microbial signatures may be potential predictive biomarkers for antidepressant response (Jiang et al., 2024).

### 6.5 Lifestyle interventions

#### 6.5.1 Exercise-mediated microbiota modulation

Physical activity is considered an effective approach for enhancing both physical and mental health, with these benefits frequently mediated through gut microbiota (Monda et al., 2017). Systematic reviews have demonstrated that regular exercise promotes microbial diversity, providing a more favorable environment for beneficial microbial populations that enhance SCFA production (Mailing et al., 2019). Furthermore, higher physical activity levels and improved cardiorespiratory fitness have been associated with increased SCFA content and enhanced fecal bacterial α-diversity (Clauss et al., 2021).

Clauss et al.’s review indicated that the microbiome of fit individuals exhibited increased butyrate production due to the increased abundances of key butyrate-producing bacterial taxa belonging to the Firmicutes phylum (Clostridiales, *Roseburia*, Lachnospiraceae, and Erysipelotrichaceae); butyrate-producing taxa as well as SCFA production have been consistently shown to increase in response to exercise (Clauss et al., 2021). Professional athletes exhibit distinctly different gut microbiota from sedentary populations, with their microbiomes predominantly composed of beneficial bacteria such as *F. prausnitzii* and characterized by higher butyrate, propionate, and acetate production levels (Mohr et al., 2020). However, high-intensity prolonged exercise may lead to increased intestinal permeability and systemic inflammation, disrupting microbial balance (Mailing et al., 2019).

#### 6.5.2 Sleep and circadian rhythms

A bidirectional regulatory relationship exists between sleep and gut microbiota. The gut-brain axis is a conduit for crosstalk between gut microbiota and the CNS, with microbial dysbiosis impairing sleep quality, and vice versa (Lin et al., 2024). Specific gut bacteria such as *Lactobacillus* and *Bifidobacterium* enhance sleep through 5-HT and GABA production, exemplifying direct microbiome influence; conversely, sleep deprivation reduces beneficial bacteria, exacerbating dysbiosis (Lin et al., 2024). Gut microbiota and their metabolites can influence circadian rhythms through neurotransmitter production, vagal signaling, and immune modulation; meanwhile, the circadian system can affect gut microbiota composition through circadian gene expression and immune responses (Li et al., 2018).

### 6.5.3 Stress management

Chronic stress is a significant risk factor for the development of psychiatric disorders, with gut microbiota playing an important regulatory role in stress responses. Wei et al. determined that CUMS can lead to gut microbiota dysbiosis in rats and induce colonic inflammatory responses, including impaired gut barrier function and elevated pro-inflammatory cytokine levels (Wei et al., 2019). SCFA supplementation can exert beneficial effects in treatment-resistant depression by modulating tryptophan metabolism, inflammation, neurotransmitters, and the MGBA (Palepu et al., 2024).

### 6.6 Traditional Chinese medicine interventions

Traditional Chinese medicine (TCM) approaches have shown preliminary promise in modulating the gut-brain axis, though the majority of supporting evidence derives from rodent CUMS models, and high-quality randomized controlled trials in humans with validated constipation-depression comorbidity diagnoses are largely absent. The findings below should therefore be interpreted as preclinical or preliminary clinical signals rather than established therapeutic evidence.

#### 6.6.1 Microbiota modulation by Chinese herbal formulas

Chinese herbal preparations may exert antidepressant-like effects by modulating neurotransmitter levels, SCFAs, BDNF, the kynurenine pathway, and pro-inflammatory cytokines (Feng et al., 2019). In CUMS mouse models, Kai-Xin-San reduced LPS and pro-inflammatory cytokine levels while increasing tight junction protein expression in the gut and BBB, ameliorating depression-like behaviors through regulation of the gut microbiota–inflammation–stress system (Cao et al., 2020). Xiaoyaosan similarly improved depression-like behavior in stressed rodents by shifting microbiota composition—reducing Firmicutes and increasing Bacteroidetes abundance—and by elevating SCFA production (Zhu et al., 2019; Qu et al., 2019; Yang et al., 2022). These mechanistic findings are consistent across studies, though their translational relevance to human comorbidity remains to be established.

#### 6.6.2 Gut-brain modulatory effects of acupuncture

Acupuncture may influence the MGBA through several mechanisms, including modulation of gut microbiota composition, restoration of intestinal mucosal and BBB integrity, vagus nerve stimulation, and attenuation of neuroinflammation (Xing et al., 2025; Xu et al., 2024). The vagus nerve is considered a critical pathway in this process, relaying gut microbial signals to the brain and contributing to mood regulation (Tan et al., 2022). However, clinical evidence specifically addressing constipation–depression comorbidity remains limited, and mechanistic conclusions are largely inferred from animal studies.

#### 6.6.3 Integrative strategies and future directions

Combining TCM with conventional antidepressants may offer synergistic benefits through complementary effects on gut microbiota composition and metabolism (Feng et al., 2019). Berberine, a single bioactive compound derived from several Chinese medicinal herbs, increased hippocampal 5-HT, NE, DA, and BDNF levels in CUMS rats while modulating SCFA metabolism via alterations in Firmicutes, Bacteroidetes, and Lachnospiraceae (Huang M. et al., 2023). These preliminary findings suggest potential value in integrative regimens, but rigorous clinical trials are needed before such approaches can be recommended for patients with constipation–depression comorbidity (Shen et al., 2023).

## 7 Challenges, limitations, and future directions

### 7.1 Methodological limitations and critical appraisal

Before discussing future directions, a critical appraisal of current evidence is warranted. First, methodological heterogeneity across microbiome studies substantially limits cross-study comparability. Many foundational studies rely on 16S rRNA amplicon sequencing, which provides taxonomic resolution only to the genus level and is subject to primer bias and database-dependent classification errors. Shotgun metagenomics offers functional resolution but remains costly and analytically complex, with few large-scale studies in constipation-depression comorbidity. Second, sample sizes in key depression-microbiota studies are frequently small (e.g., *n* = 23 in Strandwitz et al.; *n* = 46 active MDD patients in Caso et al.), limiting statistical power and generalizability. Third, confounding factors are pervasive and inconsistently controlled: dietary patterns, BMI, concurrent antidepressant or laxative use, smoking, and physical activity all independently alter gut microbiota composition and are rarely comprehensively adjusted for in the same study. Fourth, reverse causality remains a fundamental concern—it is unclear whether dysbiosis precedes and promotes comorbidity, or whether the physiological consequences of constipation and depression (reduced motility, altered diet, medication use, stress) drive observed microbiota changes. The 2024 Mendelian randomization study provides some causal inference for the depression→constipation direction, but equivalent genetic instrument analyses for microbiota-disease causality remain limited by the low heritability of most microbial taxa. Fifth, publication bias likely inflates positive findings, as null or inconsistent microbiota results are underreported. Finally, animal-to-human translational limitations are significant: germ-free mouse models and CUMS paradigms involve physiological conditions that do not faithfully recapitulate human psychiatric or functional gastrointestinal disorders, and microbiota transplant experiments in rodents cannot be directly extrapolated to clinical practice.

### 7.2 Causal inference and study design

Establishing definitive causal relationships between gut microbiota and psychiatric disorders remains a central challenge. The predominance of cross-sectional designs precludes causal inference and fails to capture temporal dynamics in microbiome composition (Eskandar, 2025). Twin cohort studies effectively control for genetic background and shared early environmental exposures, yet are similarly constrained by their cross-sectional nature (Delanote et al., 2024). Fecal microbiota transplantation (FMT) experiments provide partial causal validation in animal models; however, translation to humans must be approached with caution. Longitudinal studies and rigorously designed randomized controlled trials are needed to establish temporal causal chains (Góralczyk-Bińkowska et al., 2022).

### 7.3 Interindividual variability and standardization

Gut microbiota composition is shaped by a complex interplay of genetic, environmental, dietary, lifestyle, and pharmacological factors, resulting in substantial interindividual variability (Ratiner et al., 2024). Even among patients sharing the same psychiatric diagnosis, patterns of microbial alteration exhibit marked heterogeneity (Rathore et al., 2025). The absence of unified standards for sample collection, preservation, and sequencing protocols further limits cross-study comparability (Huang G. et al., 2023). Key parameters of microbiota-targeted interventions—including probiotic dosage, treatment duration, and strain selection for both probiotics and FMT—require systematic optimization (Xiong et al., 2023).

### 7.4 Biomarker development

Although specific taxa such as *Faecalibacterium prausnitzii* have been reproducibly associated with mood and quality-of-life outcomes across independent cohorts, these associations do not constitute validated clinical biomarkers. Clinical translation requires prospective longitudinal validation, demonstration of diagnostic performance independent of established confounders, and standardized assay protocols—prerequisites that have not yet been met for any gut microbial marker in constipation-depression comorbidity. Microbiota-based diagnostic systems therefore remain a research objective rather than an established clinical tool.

### 7.5 Precision microbiomics and emerging approaches

The integration of high-throughput sequencing, multi-omics platforms, and artificial intelligence offers new opportunities for the precise characterization of host-specific microbiota and microbe–host interactions (Ratiner et al., 2024; Feng et al., 2020). Multi-omics approaches—encompassing metagenomics, metabolomics, transcriptomics, and proteomics—represent a key strategy for elucidating gut-brain axis mechanisms, with short-chain fatty acids, tryptophan metabolites, and bile acids emerging as metabolite classes of particular mechanistic interest (Góralczyk-Bińkowska et al., 2022). Machine learning is accelerating the identification and functional characterization of microbial biomarkers and has facilitated the development of disease prediction models based on metagenomic signatures (Angelova et al., 2023).

### 7.6 Clinical translation and safety

Drawing on precision medicine frameworks established in inflammatory bowel disease, individualized therapeutic strategies informed by patients’ microbiota profiles, metabolic phenotypes, and genetic backgrounds hold considerable promise (Mousa and Al Ali, 2024). Next-generation microbial therapeutics—including defined bacterial consortia, engineered probiotics, and targeted metabolite therapies—are expanding the therapeutic landscape (O’Toole et al., 2017). Standardized long-term follow-up systems are essential for evaluating the durability of microbiota-based interventions. Rigorous safety monitoring, particularly long-term risk assessment for invasive procedures such as FMT, and strengthened regulatory oversight are requisite conditions for the safe and effective clinical implementation of microbial therapeutic products (O’Toole et al., 2017; Keller et al., 2021).

## 8 Conclusion

The gut microbiota is a critical hub connecting physiological and psychological health, and is irreplaceable in the pathogenesis, diagnosis, and treatment of chronic constipation-depression comorbidity. This review systematically summarized the multidimensional communication mechanisms of the gut–brain axis, including complex interactions among neurotransmitter metabolism, immune-inflammatory responses, HPA axis regulation, and metabolite signaling pathways. Substantial evidence has demonstrated that dysbiosis mediates the reciprocal influence between constipation and depressive symptoms through inflammatory activation, neurotransmitter imbalance, and metabolic dysregulation, forming a vicious cycle. From an interventional perspective, diversified strategies, including psychobiotics, FMT, dietary modification, and exercise provide abundant options for clinical practice. However, challenges (interindividual variability, difficulties in establishing causal relationships, and lack of standardization) remain. The multi-omics integration paradigm established by large-scale cohort studies such as the Human Microbiome Project, combined with artificial intelligence and biomarker development, has charted the direction toward individualized precision treatment (Integrative Hmp (iHMP) Research Network Consortium, 2019). The proposal of innovative concepts such as core probiotic-targeted therapy marks the transition of microbial therapeutics from empirical exploration to precision targeting (Fang et al., 2025). Looking forward, with sequencing technology advances, computational method innovations, and interdisciplinary collaboration strengthening will facilitate the development of gut microbiota modulation as an important therapeutic pillar for functional gastrointestinal disorders with comorbid psychiatric conditions (Margolis et al., 2021), providing a solid scientific foundation for achieving the integrative medicine goal of “simultaneous treatment of body and mind.”
